# Using long and linked reads to improve an Atlantic herring (*Clupea harengus*) genome assembly

**DOI:** 10.1038/s41598-019-54151-9

**Published:** 2019-11-27

**Authors:** Sunnvør í Kongsstovu, Svein-Ole Mikalsen, Eydna í Homrum, Jan Arge Jacobsen, Paul Flicek, Hans Atli Dahl

**Affiliations:** 1 Amplexa Genetics A/S, Hoyvíksvegur 51, FO-100 Tórshavn, Faroe Islands; 2 University of the Faroe Islands, Department of Science and Technology, Vestara Bryggja 15, FO-100 Tórshavn, Faroe Islands; 3 Faroe Marine Research Institute, Nóatún 1, FO-100 Tórshavn, Faroe Islands; 4 European Molecular Biology Laboratory, European Bioinformatics Institute, Wellcome Genome Campus, Hinxton, Cambridge CB10 1SD UK

**Keywords:** Genome, Ichthyology

## Abstract

Atlantic herring (*Clupea harengus*) is one of the most abundant fish species in the world. It is an important economical and nutritional resource, as well as a crucial part of the North Atlantic ecosystem. In 2016, a draft herring genome assembly was published. Being a species of such importance, we sought to independently verify and potentially improve the herring genome assembly. We sequenced the herring genome generating paired-end, mate-pair, linked and long reads. Three assembly versions of the herring genome were generated based on a *de novo* assembly (A1), which was scaffolded using linked and long reads (A2) and then merged with the previously published assembly (A3). The resulting assemblies were compared using parameters describing the size, fragmentation, correctness, and completeness of the assemblies. Results showed that the A2 assembly was less fragmented, more complete and more correct than A1. A3 showed improvement in fragmentation and correctness compared with A2 and the published assembly but was slightly less complete than the published assembly. Thus, we here confirmed the previously published herring assembly, and made improvements by further scaffolding the assembly and removing low-quality sequences using linked and long reads and merging of assemblies.

## Introduction

Atlantic herring (*Clupea harengus*) is one of the most abundant fish species in the world and is an important economical and nutritional resource. In 2016, a total of 1,639,760 tons of Atlantic herring were fished worldwide^1^. Herring is especially crucial to the Faroe Islands, where 108,244 tons were fished in 2017, constituting 7.5% of the total value of exported goods that year^2^.

The species is a pelagic, highly migratory fish, with a vast geographical distribution. Several populations of Atlantic herring have been identified, spawning in different seasons and sites in the North Atlantic Ocean^3^. Some of the populations mix to a varying degree during their feeding migrations and are only distinguished by morphological, physiological, and biological characteristics, which can be open to interpretations^4^. Identifying populations and the extent of mixed fisheries is vital to keep the fisheries sustainable. Thus, knowledge of the population structure is necessary. Disregard of population structure in fisheries management can lead to overexploitation and result in the loss of genetic variation^5^, which may be vital for adaptation in an ocean affected by climate change. Furthermore, knowledge of the population structure can be used to forensically identify fish and fish products throughout the food processing chain, and it assists in the fight against illegal, unreported, and unregulated (IUU) fishing. Genetics is a useful tool in the fight against IUU, as shown in Nielsen *et al*.^6^. Most of the commercially fished species are not model organisms, and therefore, limited genetic information is available for them. A few studies have been performed on herring population genetics, but the ability to distinguish some of the subpopulations has only been partially accomplished^4,7,8^. The availability of the assembled genome for the species in question is the ultimate basis for developing population genetic markers, to be able to map microsatellites, single nucleotide polymorphisms (SNPs), and other polymorphisms. Generally, more variations are expected in the noncoding regions than in coding regions. Therefore, assembling the whole genome rather than just the transcriptome means that more detailed population genetic markers can be developed, increasing the power for separating closely related populations.

The size of the herring genome is estimated to be approximately 850 megabases (Mb), and it consists of 26 pairs of chromosomes^9–12^. In 2016, Martinez Barrio *et al*. published the first draft of the herring genome^13^. The assembled size was 808 Mb, arranged in 73,682 contigs and 6,915 scaffolds, with a scaffold N50 of 1,860 kilobases (kb). Studies have shown that different assembly approaches may yield different assembly results^14–16^. Furthermore, combining several sequencing technologies can improve genome assemblies^17–19^. Thus, being a species of such ecological, economical, and nutritional importance, we undertook a second assembly using a different combination of sequencing technologies to verify and improve the herring genome assembly and obtain more definitive genomic information of this species. This knowledge is critical for the further study of the herring population structure and genetic variation.

Here, we sequenced the herring genome on an Illumina platform, generating paired-end, mate-pair, and linked (10x Genomics) reads. Long reads were also generated using the Oxford Nanopore Technologies platform, MinION. A *de novo* herring genome was assembled from the short reads and scaffolded using the long and linked-read data. In the last stage, our assembly was merged with the previously published assembly by Martinez Barrio *et al*.^13^ (GCF_000966335.1_ASM96633v1; here referred to as the published draft assembly) to create a more accurate genome assembly, shown by comparing the assemblies with multiple quality parameters.

## Results

### Sequencing and assembly

A paired-end library and two mate-pair libraries (both approximately 2 kb when investigated bioinformatically) were sequenced along with long (MinION) and linked (10x Genomics) reads. The same individual was sequenced with Illumina technology and on one MinION run. However, the DNA from this individual was too degraded to obtain long reads. Therefore, three additional MinION runs were performed using a fresh sample from a second individual, which resulted in longer reads and higher output. The total output for the four runs was 985,281 reads with an N50 of 8,119 bp. A third individual was sequenced using 10x Genomics technology, to obtain input fragments that were as long as possible. Table 1 presents a summary of the sequencing results.

**Table 1:** Summary of sequencing results.

Sequencing technology and library type	Raw reads		Reads after QC		Coverage after QC
	No. of reads	Bases ≥ Q30	No. of reads	Bases ≥ Q30	
Illumina - Paired end	668,361,981	78.5%	490,582,474	91.1%	150.0x
Illumina - Mate pair with insert size 4.5 kb*	591,526,598	68.1%	156,135,780	90.4%	26.3x
Illumina - Mate pair with insert size 7 kb*	116,602,405	79.2%	44,016,755	94.4%	8.8x
Illumina - 10x Genomics	363,163,358	61.3%	—	—	78.5x
MinION	1,135,273	—	985,281	—	2.4x

Coverage refers to the coverage of the estimated 850 Mb Atlantic herring genome. Quality control (QC) for paired-end data consisted of quality trimming and adapter removal. QC for mate-pair data consisted of quality trimming and sorting of reads based on presence of adapter in reads. No QC was performed on the 10x Genomics reads as recommended by 10x Genomics. The QC for the MinION reads consisted of alignment to the draft assembly and only aligned reads longer than 500 bp were kept.

*When the mate pair library data were investigated bioinformatically, both libraries seemed to have an insert size of 2 kb.

To generate an improved herring genome assembly, we first generated *de novo* assemblies from the short-read data using the AllPaths-LG and SGA assemblers^20,21^ with different parameters (Supplementary Table S1). The assembly with the best summary statistics (*i*.*e*., number of contigs, number of scaffolds, and N50) was named A1. This assembly was improved using gap closing software and long and linked reads for scaffolding (see Materials and Methods) resulting in the A2 assembly. Lastly, the A2 assembly was merged with the published draft assembly to obtain the best assembly possible (A3). Table 2 presents the characteristics of these assemblies. For comparison, we generated an alternative assembly using the hybrid assembler, MaSuRCA, which Zimin *et al*.^22^ claimed to have equal or superior performance to AllPaths-LG. This resulted in a highly fragmented assembly (74,436 scaffolds and N50 of 28 kb). Thus, in our hands, MaSuRCA did not perform better than AllPaths-LG combined with SSPACE-LongRead^23^ and ARCS^24^. The MaSuRCA assembly was not further used in this study.

**Table 2:** Comparison of assemblies A1, A2 and A3 from this study and the published draft assembly.

Metric	A1	A2	A3	Draft
# scaffolds (>= 0 bp)	15,378	9,444	2,419	6,915
# scaffolds (>= 1,000 bp)	15,188	9,334	2,419	6,915
# scaffolds (>= 5,000 bp)	10,057	6,348	1,709	2,267
# scaffolds (>= 10,000 bp)	8,049	5,378	1,573	1,964
# scaffolds (>= 25,000 bp)	5,166	3,798	1,319	1,481
# scaffolds (>= 50,000 bp)	3,252	2,678	1,043	1,131
Total length of scaffolds (>= 0 bp)	702,694,152	729,318,454	790,426,535	807,711,962
Largest scaffold (bp)	2,291,227	3,948,801	13,043,132	13,053,552
Scaffold N50 (bp)	177,425	332,253	1,971,137	1,897,858
# contigs (>= 0 bp)	131,323	112,927	61,451	67,061
Total length of contigs (>= 0 bp)	524,819,960	551,688,118	711,593,948	725,034,955
Largest contig (bp)	169,324	179,560	251,421	245,657
Contig N50 (bp)	6,450	8,441	25,590	25,381
GC (%)	43.07	43.06	44.13	44.11
# Ns per 100 kbp	25,665	24,588	9,995	10,314
# predicted rRNA genes	52 + 15 part	60 + 12 part	57 + 10 part	57 + 10 part

Results from the QUAST analysis, all statistics are based on contigs of size >= 3,000 bp, unless otherwise noted; for example, # contigs (>= 0 bp) includes all contigs.

### Did scaffolding with linked and long reads improve the assembly?

To assess the level of improvement obtained through gap-closing and scaffolding with long and linked reads, we compared the assemblies using QUAST^25^. QUAST is a tool for assessing the quality of genome assemblies and can be used both with and without a reference assembly. Without a reference assembly, QUAST calculates several descriptive summary statistics for the assemblies, which are mostly based on the size and fragmentation of the assemblies (*e*.*g*., the number of scaffolds, length of the assembly, N50, and NG50). GC content, Ns per 100 kbp, and predicted rRNA genes are also found by QUAST. Table 2 presents selected QUAST results, and as expected, both the fragmentation and size of the assembly were improved when A1 was scaffolded with long and linked reads, resulting in A2. The number of scaffolds decreased by roughly 38%; both the total length and length of the largest scaffold increased and N50 almost doubled (Table 2). The same trend could be seen in the number and length of contigs. Moreover, the completeness of the assembly improved, and Ns per 100 kbp decreased by 1,077. There were 60 complete rRNA genes in A2, compared with 52 in A1, and 12 partials in A2 compared with 15 in A1 (Table 2).

The completeness of the assemblies was further assessed using Benchmarking Universal Single-Copy Orthologs (BUSCO), which searches for near-universal single-copy orthologs based on evolutionarily-informed expectations of gene content^26^. Different BUSCO sets are used for different groups of organisms, and presently the set for ray-finned fish includes 4,584 genes. The BUSCO analysis showed the same trend as the QUAST analysis when progressing from assembly A1 to A2. The number of complete BUSCOs increased by 251, fragmented BUSCOs decreased by 172, and missing BUSCOs decreased by 79 (Table 3), indicating a more complete assembly.

**Table 3:** Benchmarking Universal Single-Copy Orthologs (BUSCO) analysis of the A1, A2, and A3 assemblies and the previously published draft herring genome assembly.

BUSCOs	A1	A2	A3	Draft
Complete BUSCOs	3,598	3,849	4,258	4,348
Complete and single-copy BUSCOs	3,473	3,706	4,085	4,176
Complete and duplicated BUSCOs	125	143	173	172
Fragmented BUSCOs	409	237	177	105
Missing BUSCOs	577	498	149	131
Total BUSCO groups searched	4,584	4,584	4,584	4,584

The summary statistics in Table 2 are commonly used metrics to compare assemblies, but they only show how fragmented the assemblies are and say little about the completeness and correctness of the assemblies. Furthermore, these traditional metrics do not necessarily indicate which assembly is of the highest quality. In fact, N50 has been shown to be negatively correlated with the quality of an assembly^27^.

**Figure 1:**
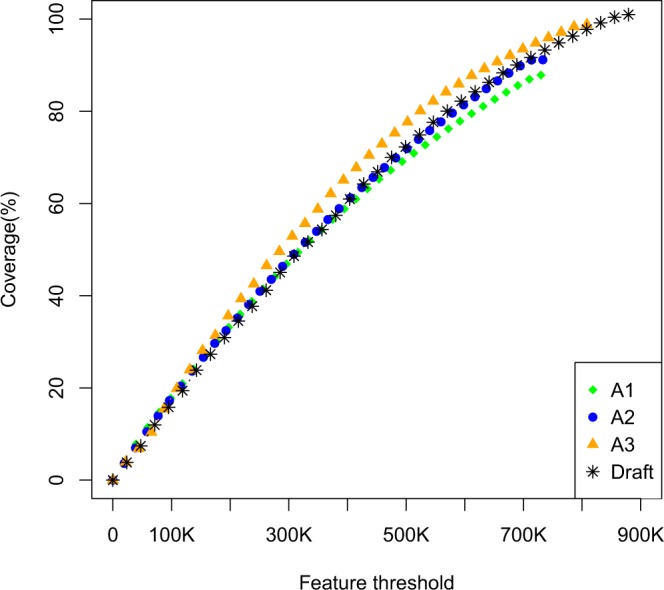
Feature response curves for the A1, A2, A3 and draft assembly. The FRCs were generated using FRC^bam[27]^ and plotted in R v3.4.3^51^.

To assess the assembly correctness, a feature response curve (FRC) was calculated for each assembly. FRC is a metric that, according to the authors Narzisi and Mishra^28^, captures the trade-offs between quality and contig size. The analysed features and underlying logics were described by Phillippy *et al*.^29^. In short, a steeper curve indicated an assembly of higher quality. The results from this comparison can be seen in Fig. 1. The FRCs for A1 and A2 diverged at a higher feature threshold, with A2 being steeper.

FRC^bam^ outputs 14 categories of features based on both paired-end and mate-paired data^27^. Features are areas on the assembly that show indications of assembly errors based on the alignment of sequencing reads. Through examination of the different features separately it became obvious that the assemblies had different types of features (*i*.*e*., different strengths and weaknesses). We ranked the assemblies for all 14 types of features so that the assembly with the steepest FRC for the specific feature obtained the best ranking (1^st^), we then summed over all the features types to obtain a ranking of the assemblies based on overall features. This ranking is shown in Table 4, and overall A2 (2^nd^) was ranked better than A1 (4^th^). FRCs for the specific features can be seen in Supplementary Figs. S1–S14. As mentioned earlier, the FRC also accounts for the contig size. However, examining only the total number of features, we saw that A1 had 564,464 features, whereas A2 had 544,122, showing a reduction of 3.6%.

**Table 4:** Ranking of the A1, A2, A3 and draft assemblies based on FRCs from 11 different feature types.

Feature type	A1	A2	A3	Draft
COMPR_MP	1^st^	2^nd^	3^rd^	4^th^
COMPR_PE	4^th^	3^rd^	1^st^	2^nd^
HIGH_COV_PE	3^rd^	4^th^	2^nd^	1^st^
HIGH_NORM_COV	3^rd^	4^th^	2^nd^	1^st^
HIGH_OUTIE_MP	3^rd^	4^th^	1^st^	2^nd^
HIGH_SPAN_MP	4^th^	1^st^	1^st^	3^rd^
HIGH_SPAN_PE	3^rd^	1^st^	2^nd^	4^th^
LOW_COV_PE	3^rd^	1^st^	4^th^	2^nd^
LOW_NORM_COV_PE	2^nd^	1^st^	4^th^	3^rd^
STRECH_MP	3^rd^	4^th^	2^nd^	1^st^
STRECH_PE	1^st^	2^nd^	3^rd^	4^th^
Sum	30	27	25	27
Overall rank	4^th^	2^nd^	1^st^	2^nd^

FRC^bam^ was used for the FRC analysis. Rank: Each of the 14 features (potential assembly errors) analysed by FRC^bam^ were individually ranked (based on Supplementary Figs. S1–S14) from 1^st^ to 4^th^, with 1^st^ having the steepest FRC. The ranks were summed without weighting the features. Feature types HIGH_OUTIE_PE, HIGH_SINGLE_MP, and HIGH_SINGLE_PE were excluded because of limited data points in the FRC. Feature types are explained in the legends of Supplementary Figs. S1–S14.

BUSCOs (Table 3) did provide an indication of the level of completeness, but we wanted to further inspect the completeness by looking at the connexin (gap junction protein) gene family. Generally, bony fish have approximately 40 recognised connexin genes^30,31^. Most of these genes have their coding sequence in a single exon, greatly facilitating a manual analysis. Additionally, these genes have two conserved regions that are easily recognised across the gene family. From other species, including different bony fish, it is known that some of these genes are located close to each other^30,32^. In this context, the conserved regions might be considered repetitive sequences, which could make these genes more prone to assembly errors.

In our manual analysis of the connexin genes we first identified 51 herring connexin genes from the draft assembly by Martinez Barrio *et al*.^13^. Of these, 49 connexin genes were already predicted and annotated by Martinez Barrio *et al*. and available in GenBank. In addition to the 49 connexin genes, one connexin gene was predicted as a *KAT6B-like* gene, and one connexin gene (called *Cx39*.*2* or *GJD2like*) was not predicted but found in our searches. Some of the genes found in the draft assembly were believed to be duplicates or triplicates, based on the 98–100% sequence identities (see Table 5 and Supplementary Table S2). Thus, these genes were either very recently duplicated or arose through erroneous assembly, and we consider 46 as a more likely number of functional connexin genes in herring. More details on the analysis of connexin genes in herring and other teleosts can be found elsewhere (Mikalsen SO, Tausen M and í Kongsstovu S, submitted).

**Table 5:** Suspected assembly errors in the connexin genes of the A1, A2, and A3 assemblies and the published draft assembly.

Connexin^a^	mRNA Acc. no^b^	A1		A2		A3		Published draft assembly	
		Scaffold^c^	Position^c^	Scaffold	Position	Scaffold	Position	Scaffold	Position
*Cx32*.*2like*	XM_012828709	—		—		38	1911178–1910384	NW_012218207	1912581–1911787
*GJA5like*	XM_012816449	—		5201	1–939	810	17809–16457	NW_012219501	17809–16457
*GJA5like*	XM_012840593	1893 1893	77725–77986^Fr^ 81019–81888^Fr^	1668 1668	85132–86034^Fr^ 81418–81660^Fr^	2	4108234–4107059	NW_012223947	4109526–4108351
*GJB3like*	XM_012818491 *XM_012818489*	1447	67762–67004	893	284447–283689	258 *258*	698572–697814 *719052*–*718294*	NW_012219726 *NW_012219726*	699040–698282 *719520*–*718762*
*GJB3like*	XM_012822385 *XM_012822374* *XM_012822365*	41	751478–750609	17	751444–750575	19 *19* *19*	2723482–2722843 *2725676*–*2724807* *2728190*–*2727321*	NW_012217989 *NW_012217989* *NW_012217989*	2733880–2733241 *2736074*–*2735205* *2738588*–*2737719*
*GJB4like*	XM_012818492 *XM_012818490*	1447	70822–70040	893	287507–286725	258 *258*	722112–721330 *701632*–*700850*	NW_012219726 *NW_012219726*	722580–721798 *702100*–*701318*
*GJD2like*	XM_012838313	1213 1213	110633–109796^Fr^ 93762–93246^Fr^	1118 *1996* *1118* *1118*	117810–116668 *74407*–*74559* *102088*–*101572* *109471*–*108955*	35 *35* *35*	1605461–1606603 *1612109*–*1612625* *1617455*–*1617970*	NW_012223366 *NW_012223366* *NW_012223366*	1605047–1606189 *1611695*–*1612211* *1617041*–*1617556*
*GJD3like*	XM_012837668 *XM_012837669*	—		—		81 81 *81* *81* *81*	1079728–1080054^e1^ 1080603–1081282^e2^ *1090945*–*1091624* *1090176*–*1090488* *1091628*–*1091757*	NW_012223169 NW_012223169 *NW_012223169* *NW_012223169* *NW_012223169*	1079728–1080054^e1^ 1080603–1081282^e2^ *1102966*–*1103278*^*e1*^ *1090945*–*1091624*^*e2*^ *1091628*–*1091757*^*e2*^
*GJD3like*	XM_012837670	4907 4907	23078–22754^e1^ 22252–21980^e2^	216 216	557978–557512^e1^ 558804–558480^e2^	81 81 *81*	1102952–1103276^e1^ 1103742–1104367^e2^ *1090162*–*1090486*	NW_012223169 NW_012223169 *NW_012223169*	1090162–1090486^e1^ 1103742–1104367^e2^ *1102952*–*1103276*

Suspected errors include regions of repetition (position written in italics) and missing connexin genes (represented as −). Fr, e1 and e2 indicate fragmented, exon 1 and exon 2, respectively.

^a^The name is an abbreviation of the name given by the mentioned GenBank accession numbers. For example, ‘GJB3-like’ should be read as ‘gap junction beta-3 protein-like, mRNA’. Please note that unique genes may have the same name.

^b^GenBank nucleotide (nr) accession numbers for predicted transcripts from the published draft assembly. If the gene had several predicted transcription variants, only transcription variant 1 was included in the analyses. If several identical, or near identical (>98%) transcripts have been predicted, the other accession numbers are given in italics.

^c^The positions here regarded as the coding sequence of the gene is given in normal font (the exon/intron borders are not exact), and the ‘suspect repeated’ regions are given in italics. The positions are given as the coding direction (*i*.*e*., from the 5′) independent of whether the sequence is on the plus or minus strand.

Furthermore, we investigated the presence of the connexins in our progressive assemblies A1, A2 and A3 (the latter is described in more details below). There were 3 connexins lacking in A1 (*Cx32*.*2like*_XM_012828709, *GJA5like*_ XM_012816449, and *GJD3like*_XM_012837668), one of which was found in A2 (*GJA5like*_ XM_012816449). In addition, the *GJD2like*_ XM_012838313 and *GJA5like*_XM_012840593 genes were fragmented in A1 (Table 5). The fragmentation of *GJA5like*_ XM_012840593 was still present in A2, whereas the *GJD2like*_ XM_012838313 was found as a single complete coding sequence in A2, but parts of the gene were now triplicated (Table 5). Thus, the duplications indicated in the published draft assembly were not present in A1 or A2. They were also not present in other bony fish, such as Japanese eel (diverged before herring), Atlantic cod (diverged after herring) or zebrafish, which is probably the most heavily investigated teleost, and is supposed to have common divergence with herring from the remaining teleosts^33,34^. As this study came to an end, a chromosome level assembly of the herring genome (GCA_900700415.1) was made available along with a preprint paper^35^. The connexin duplications were also absent in this new assembly. Thus, we consider it likely that these duplications are caused by erroneous assembly.

### Merging the assembly from this study with the draft assembly

Even though A2 was an improvement on A1, it was shorter and more fragmented than the published draft assembly as well as less complete (Tables 2 and 3). To generate the best possible herring assembly from the available data, A2 and the previously published assembly were merged, giving rise to A3. As can be seen in Table 2, A3 had fewer scaffolds (2,419 compared with 6,915), higher N50 (1,971,137 compared with 1,897,858), and 319 fewer Ns per 100 kbp than the draft assembly. Nevertheless, the largest scaffold was slightly shorter in A3, and there were fewer complete BUSCOs (4,258 compared to 4,348) and more fragmented BUSCOs (177 compared to 105) in A3 compared to the draft assembly (Tables 2 and 3). In addition, the total length of A3 was 17 Mb shorter than the total length of the previously published draft assembly; 3 Mb of this difference was explained by the decrease in gap length. The Metassemble^36^ software package was used for merging the two assemblies. In short, the software aligns the assemblies and confirms the merging steps via mate-pair reads. In addition, unaligned sequences are removed. In the case of A3, approximately 10 Mb of sequences (3,912 short scaffolds from the draft assembly) were removed, which was the main reason for A3 being shorter than the draft assembly. Removal of these short scaffolds was another reason why the summary statistics improved. Another partial explanation was that some areas were accidentally (and probably wrongly) repeated in the draft assembly but resolved in A3. Nevertheless, 103 breakpoints and 3,224 insertions were introduced in the generation of the A3 assembly. In addition to the removal of the 3,912 short scaffolds, 202 scaffolds were joined to form 101 scaffolds. To test if the removal of scaffolds was the only reason why the summary statistics improved, the removed scaffolds were added to A3 and the summary statistics for this combined assembly were calculated. This assembly had 6,331 scaffolds, an N50 of 1.96 Mb, a total length of 800 Mb, and a gap length of 80 Mb, indicating that the removed scaffolds did contribute to the improvements in the summary statistics but were not the sole reason.

The FRCs for A2 and the draft assembly were highly similar, and the main difference was the total length of the assemblies. A2 was shorter than the draft assembly, and thus the FRC only reached 91% coverage (Fig. 1). A3 showed a steeper FRC than the draft assembly but was slightly shorter. When ranking the assemblies based on overall features, the merged A3 was ranked as 1^st^, whereas the published draft assembly and A2 were ranked 2^nd^. The total number of features improved with the merging of the assemblies from 544,122 in A2 and 487,486 in the draft assembly to 473,588 in A3. These results indicate that A3 is more correct than A2 and the draft assembly.

The connexin analysis revealed duplications or triplications in six connexin genes in both A3 and the draft assembly. The same duplications/triplications were present in A3 and the draft (Table 5), suggesting that both these assemblies have some issues with repeats. Nevertheless, the missing connexins in A1 and A2 were present in both A3 and the draft.

**Figure 2:**
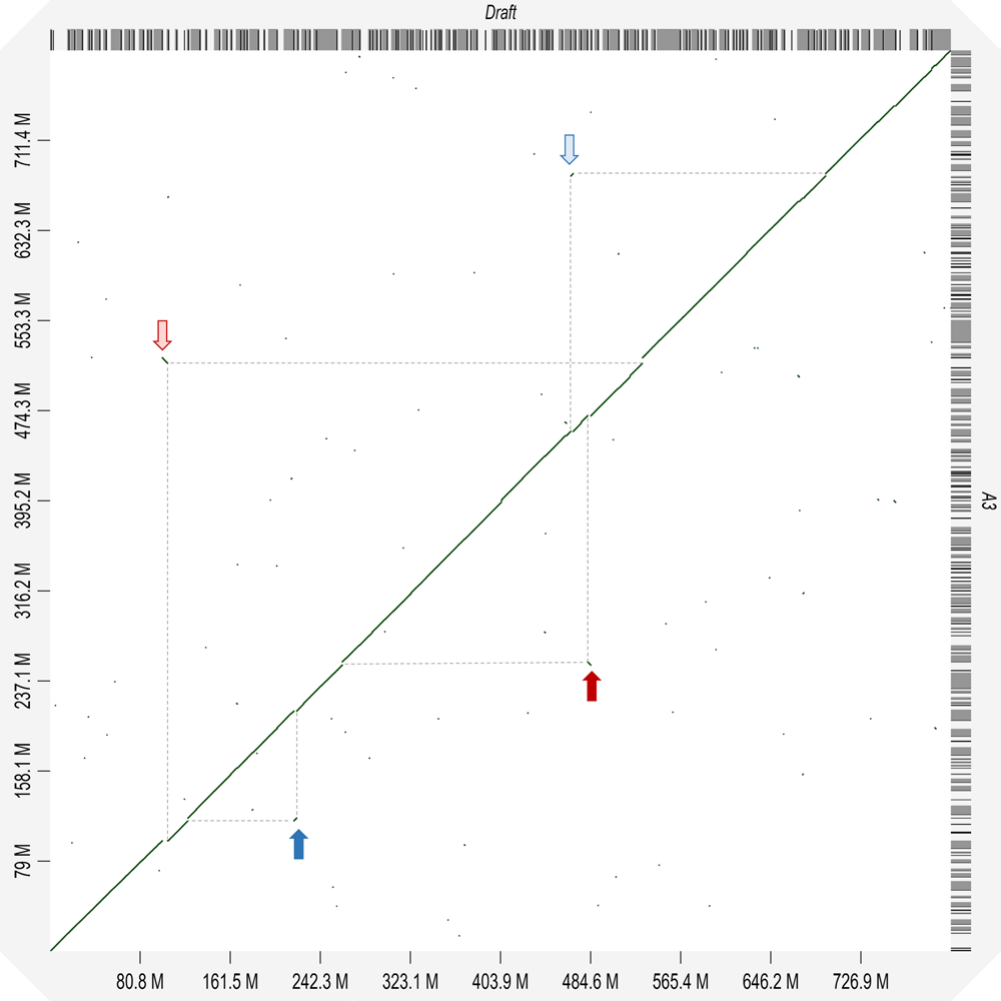
Dotplot showing the whole genome alignment between the published draft herring genome assembly and the A3 herring genome assembly from this study. The alignment was generated using D-Genies^37^. Examples of transpositions between the two assemblies are indicated by blue arrows and examples of inverted transpositions are shown by red arrows. The horizontal and vertical grey dotted lines indicate the positions on the two assembles that are affected.

Whole genome alignments were generated using the web tool D-Genies to investigate whether any major structural variations existed between the assemblies^37^. Figure 2 shows the alignment between A3 and the published draft assembly. The largest rearrangements are indicated by the coloured arrows, and our notion is that these indicate some of the improvements made by the merging of the assemblies.

As mentioned, a herring chromosome level assembly became available very recently^35^. A QUAST run with this assembly as a reference was conducted to compare the all available assemblies. Table 6 lists selected QUAST results. It was evident that the A2 assembly had the most misassemblies whereas A1 has the fewest, indicating that the scaffolding steps caused several misassemblies (Table 6). It was also evident that low-quality sequences were removed in A3 because A3 had the fewest number of misassembled scaffolds, fewest unaligned scaffolds, lowest duplication ratio, and longest alignment. Some of these misassemblies might be individual variations rather than actual misasseblies. However, the chromosome level assembly had 4,036 complete, 3,881 complete and single copy, 155 complete and duplicated, 174 fragmented and 374 missing BUSCOs. This indicated that the chromosome level assembly was less complete than both the previously published draft assembly and A3.

**Table 6:** QUAST generated comparisons of A1, A2, and A3 assemblies and the published draft herring assembly, using the new chromosome level assembly as reference.

Metric	A1	A2	A3	Draft
# misassemblies	4,284	8,810	6,045	6,034
# misassembled scaffolds	2,306	2,499	572	649
Misassembled scaffolds length (bp)	326,883,342	549,092,805	621,722,316	616,769,397
# local misassemblies	19,581	30,042	55,799	55,990
#scaffold gap extensive misassemblies	369	806	436	439
# scaffold gap local misassemblies	82,670	70,490	23,309	22,741
# possible misassemblies by TEs	2,922	4,056	3,640	3,548
# unaligned misassembled scaffolds	1,292	950	892	1,157
# unaligned scaffolds (full + partial)	463 + 8,064	247 + 5,563	61 + 1,706	228 + 2,256
Unaligned length (bp)	84,214,870	97,034,653	211,181,030	217,841,770
Genome fraction (%)	59.41	61.11	66.87	66.94
Duplication ratio	1.42	1.42	1.19	1.20
# mismatches per 100 kbp	709.66	883.77	1,634.08	1,643.42
# indels per 100 kbp	110.22	109.28	127.10	127.13
Largest alignment (bp)	1,320,028	1,496,625	1,700,060	1,587,972
Total aligned length (bp)	435,132,440	456,232,649	501,922,313	503,353,702
NA50 (bp)	30,242	35,372	77,322	69,158
NGA50 (bp)	23,530	35,287	114,419	112,940
LA50 (bp)	3,156	2,771	1,498	1,600
LGA50 (bp)	3,717	2,775	1,159	1,174
K-mer-based compl. (%)	42.13	43.00	51.92	51.95
K-mer-based correct length (%)	72.39	39.82	54.43	57.40
K-mer-based misassembled length (%)	19.02	54.57	43.06	38.94
# k-mer-based misjoins	800	1,967	433	423

Thus, all results are relative to the chromosome level assembly.

## Discussion

In this study, we generated a *de novo* assembly of the herring genome and improved its fragmentation, correctness, and completeness with gap closing software and long and linked reads. The assembly was then combined with the published draft assembly^13^, resulting in a less fragmented assembly that was slightly less complete but overall showed an increase in correctness, based on summary statistics, BUSCO, connexin, and FRC analyses.

Comparing two or more assemblies is not necessarily straightforward. Simple summary statistics exist, such as the number of contigs/scaffolds, N50, L50, and total assembly length. However, these metrics only evaluate the size and fragmentation; but they say very little about the quality or correctness. Studies have compared several assemblies, such as Assemblathon 1, Assemblathon 2 and GAGE^14–16^, and these studies have used several metrics to get a fair comparison. A common conclusion has been that using only one metric to evaluate assemblies does not necessarily reveal the optimal assembly. Different metrics indicate different strengths and weaknesses of assemblies. We therefore chose to use several different metrics, which we believe appropriately represents the quality of the assemblies, to compare the assemblies in this study.

A comparison of A1 and A2 revealed that the long and linked reads improved the fragmentation of the assembly. The number of scaffolds decreased by 38% while the N50 almost doubled, but the gap length increased slightly. This increase in gap size was to be expected from this scaffolding step, because SSPACE-LongRead does not include the MinION read in the assembly^23^. The number and length of contigs also improved, with 18,396 fewer contigs and 26 Mb longer total contig length (Table 2) . The completeness of the assembly was also improved with scaffolding. A2 had fewer Ns per 100 kbp, an increased number of complete BUSCOs, a decreased number of fragmented BUSCOs, and increased complete predicted rRNA genes (Tables 2 and 3). Furthermore, the correctness improved. The number of total features in A2 decreased and the A2 FRC was improved. In addition, a missing connexin gene in A1 was present in A2 but new duplications in other connexin genes were introduced (Table 5). These results, as well as recent *de novo* assemblies of fish genomes^19,38^ and genomes from other organisms^18,39,40^, illustrate that long-read technology is highly useful in *de novo* genome assemblies.

A comparison of A2, A3 and the previously published draft assembly revealed the A3 assembly to have the best summary statistics (Table 2). Some of this improvement was because of the removed scaffolds in the merging step, but as mentioned above, even when these scaffolds were included, the summary statistics were superior to those of the draft assembly. A3 also had the fewest total features; however, the draft assembly had slightly higher level of completeness compared with A3 (4,348 complete BUSCOs compared to 4,258; Table 3). A3 was also shorter than the draft assembly. This trend of an improved versions of an assembly showing shorter assembly length was also seen in the improved cod assembly published by Tørresen *et al*.^41^. Furthermore, Holt *et al*.^42^ found fewer predicted coding genes in the improved pigeon genome even though the increases in N50 and N90 were more pronounced than in the present study. The FRC for A3 was steeper than the A1, A2, and draft assembly FRCs (Fig. 1). In relation to the connexin genes, the A3 assembly had the same repeat issues as in the draft assembly (Table 5). In summary, merging A2 and the previously published assembly resulted in a mostly improved assembly, although problems probably still remain with incomplete coverage and duplications.

The A3 assembly only consists of sequences supported by alignment between A2, the draft assembly, and sequencing reads. The A3 assembly constitutes nearly 90% of the estimated herring genome^9–12^. In other words, the A3 assembly is a highly accurate and validated version of the herring genome in the sense that it highlights the regions and their accuracies found by different sequencing technologies and different assemblers. In recent years, the problem of reproducibility has been highlighted and much discussed^43^. Here, we were able to confirm the majority of the published herring genome assembly using different wet lab and *in silico* approaches, as well as generated an improved assembly that we can have strong confidence in. Nevertheless, the A3 assembly is based on four different herring individuals. Generating a genome assembly from several individuals might result in poorer assembly results because the individual variations (*e*.*g*., structural rearrangements or microsatellites) may complicate the assembly process. Comparing assemblies based on different individuals is also challenging, because it might not be possible to tell if assembly differences are due to individual variation or assembly error. Using a single individual to generate an assembly is therefore preferable, but due to degraded DNA this was not possible in this study. This means that some of the corrections and differences found in this study could be individual differences between the herring used in this study and the one used by Martinez Barrio *et al*.^13^. Thus, the A3 assembly approaches an average herring genome rather than a genome from a specific herring.

As mentioned earlier, a high-quality chromosome level assembly of the herring genome was made available just as this study was coming to an end. We found that all the available assemblies had misassembly issues compared with the new chromosome level assembly. A1 had the fewest misassemblies, whereas A3 had the fewest misassembled scaffolds relative to the chromosome-level assembly. From this comparison, it was evident that scaffolding using linked and long reads can cause misassemblies. However, using more stringent scaffolding parameters and more data would reduce the number of misassemblies introduced. As mentioned above, some of these misassemblies could also be variations between the individuals used for the various assemblies and not true misassemblies. A3 and the published draft assembly were highly similar in this comparison. A3 had fewer misassembled scaffolds, fewer local misassemblies, fewer unaligned scaffolds (both full and partial alignments), shorter unaligned length, slightly lower duplication rate, the largest alignment, and higher NA50 and NGA50. By contrast, the draft assembly had fewer misassemblies, shorter misassembled scaffold length, a slightly higher fraction of the genome assembled, and a longer total aligned length (Table 6). It is also worth mentioning that the BUSCO analysis revealed both the A3 and draft assemblies to be more complete than the chromosome level assembly, at least in relation to the number of genes.

To conclude, the A3 assembly was the most complete and correct herring genome assembly with the best summary statistics. This assembly is an improvement on the previously published herring draft genome assembly in terms of correctness, and acts as a validation of the herring genome assembly. The results from this study underline how important long and linked read data are in *de novo* genome assembly. Both the long and linked reads improved the herring genome assembly in this study. Combining the assemblies from this study with the draft herring assembly resulted in an improved herring genome assembly. Additionally, this study showed, in agreement with previous studies^14–16^, the importance of comparing both the correctness and completeness of genome assemblies.

## Materials and Methods

### Sample collection and DNA extraction

A single Atlantic herring kidney sample was sequenced on a NextSeq500 sequencer (Illumina, San Diego, California, United States) and a MinION nanopore sequencer (Oxford Nanopore Technologies, Oxford, England). The herring was collected on a research cruise by the Faroe Marine Research Institute in the summer of 2015. The kidney sample was stored in RNAlater (ThermoFisher Scientific, Waltham, Massachusetts, United States). After 24 hours at room temperature the sample was frozen until used. DNA was extracted using an AS1000 Maxwell 16 instrument (Promega, Madison, Wisconsin, United States) and the Maxwell 16 Tissue DNA purification kit (Promega). DNA concentration was measured using a Qubit 3.0 fluorometer (ThermoFisher Scientific).

The sample for another three MinION runs was caught in Haraldssund, Faroe Islands, by the local fishing boat ‘Sildin’. In an attempt to obtain DNA molecules as long as possible, the DNA was extracted as soon as the boat came ashore. It was extracted from the kidney using an AS1000 Maxwell 16 instrument and the Maxwell 16 Tissue DNA purification kit. The smaller DNA fragments were excluded by a 0.8x volume of AMPureXP bead (Beckman Coulter, Brea, California, United States) clean-up, as per the manufacturer’s instructions. DNA concentration was measured using the Qubit 3.0 fluorometer and the purity was measured using a NanoPhotometer™ Pearl instrument (IMPLEN, Munich, Germany).

The sample used for 10x Genomics sequencing was caught by the local fishing boat ‘Grani’ on Kaldbaksfjørður, Faroe Islands. The DNA from the kidney was extracted using the MagAttract HMW DNA Kit (Qiagen, Hilden, Germany).

### Ethics

The herring samples were received from stock assessment cruises and commercial catches. No fish were caught for the purpose of this project, and all fish were dead when they were selected. Thus, no ethical approval was required.

### Library preparation for Illumina sequencing

For the paired-end sequencing, the DNA was fragmented to roughly 300 bp using a Covaris M220 focused-ultrasonicator (Covaris, Woburn, Massachusetts, United States). The library was then prepared using the KAPA LTP Library Preparation Kit (KAPABiosystems, Wilmington, Massachusetts, United States) and quantified using the KAPA Library Quantification Kit (KAPABiosystems), following the manufacturer’s instructions. The paired-end library was sequenced on a NextSeq500 (Illumina) using one Mid and one High Output v2 kit.

Two mate-pair libraries, with intended insert sizes of 4,500 bp and 7,000 bp, were prepared using the Nextera Mate-Pair Library Preparation kit (Illumina), following the manufacturer’s instructions. The mate-pair libraries were quantified using the KAPA Library Quantification Kit (KAPABiosystems) and sequenced on a NextSeq500 (Illumina). However, when later investigated bioinformatically, both libraries seemed to have an insert size of approximately 2 kbp. This was most likely because of error in the library preparation and/or fragmented DNA. One of the libraries was sequenced with a High Output v2 kit while the other was sequenced with a Mid Output v2 kit.

### Oxford nanopore technologies

Four different MinION runs were conducted. The library for the first run was prepared using the same DNA sample as the Illumina sequencing together with the Rapid Sequencing kit (SQK-RAD001). The library was sequenced on a FLO-MIN105 flow cell and run for 48 hours. After the run, the reads were uploaded to Metrichor v1.2.6 for base calling. To obtain longer reads, a fresh DNA sample from a different individual was used for the subsequent MinION runs. Run two was conducted by using the Rapid Sequencing kit (SQK-RAD002) and a FLO-MIN107 flow cell. The MinION ran for 28 hours and reads were uploaded to Metrichor v1.5.7 for base calling. Runs three and four were conducted using the Ligation Sequencing kit (SQK-LSK108) and FLO-MIN107 flow cells. The MinION ran for 48 hours and the reads were base-called using Albacore v1.2.5 (Oxford Nanopore Technologies). All protocols followed the manufacturers’ instructions, except for the SQK-LSK108 kit where the DNA repair step was omitted.

### 10x Genomics

The linked reads were generated from a 10x Genomics library prepared by the Chromium Genome Reagent Kit (10x Genomics, San Francisco, California, United States) according to the manufacturer’s instructions and altered according to the technical note ‘Guidelines for De Novo Assembly of Genomes Smaller than ~3 Gb using 10x Genomics® Supernova TM V1.2’^44^ and personal communication with 10x Genomics staff. The library was sequenced on a NextSeq. 500 (Illumina) using a High Output v2 kit.

### Data pre-processing

All the data processing, assemblies and comparisons were performed on the EMBL-EBI cluster in Hinxton, except for the manual connexin gene analysis.

Trimmomatic v0.36 was used to remove adapter sequences and trim low-quality bases with an average quality score lower than 20 (sliding window of four bases) from the paired-end data^45^. Then, AfterQC v0.4.0 was used to remove the polyG reads^46^. The mate-pair data were also subjected to the same trimming conditions as the paired-end data using Trimmomatic, but adapters were not trimmed. In addition, the data were also processed using NextClip v1.3.1, and only the reads with one or both adapter sequences were used^47^.

FastQC v0.11.5 was used to assess the quality of all the sequencing data^48^. Poretools v0.6.0^49^ was used to extract the FASTQ files longer than 500 bp from MinION runs one and two, whereas Albacore v1.2.5 was used for runs three and four.

### The assembly process

The first assembly (A1) was generated using the Illumina data and the de Bruijn graph assembler AllPaths-LG v52488^20^. This assembler was chosen because of the size of the genome and the results from the Assemblathon 2 study^16^, where it performed well on the fish genome assembly. The Illumina data were generated with this assembler in mind. Several parameters and subsets of the data were tested, and the best assembly was chosen for further use in this study. In addition, the SGA v0.10.15^21^ and MaSuRCA v3.2.2^22^ assemblers were tested, but did not yield as good assemblies as AllPaths-LG assembler. Supplementary Table S1 contains the different parameters and subsets of the data used for the different assembly runs.

A2 was generated by closing gaps in A1, in addition to two scaffolding steps. The GapFiller v1.10 software package was used to close gaps. In short, this software aligns sequencing reads to the assembly and then tries to extend the ends of the contigs, if enough sequencing reads support this^50^. We ran this software for 20 iterations. The resulting assembly was then scaffolded with four runs of MinION reads using the SSPACE-LongRead v1.1 software package^23^. In addition to the default parameters, the options −a 500 and −l 1 were used, indicating the length of alignment and number of links required for scaffolding. The linked reads were intended for a *de novo* assembly using the Supernova v1.2.2 assembler (10x Genomics) but because of a problematic sequencing run the data did not yield a good assembly (results not shown). Therefore, a second scaffolding step was performed using the linked reads and ARCS v1.0.5^24^ (default parameters). Simply stated, ARCS and SSPACE-LongRead scaffold sequences by aligning the new data (linked and long reads, respectively) to the sequences (A1 in our case) and if these new data align to different sequences these are merged^23,24^.

A3 was generated by combining A2 and the draft assembly using Metassembler v1.5^36^. The previously published draft assembly was used as the primary assembly, together with the mate-pair data from this study. A run with A2 as the primary assembly was also conducted but resulted in a poorer assembly. The merged assembly was again scaffolded using the linked reads and ARCS, as described above.

### Comparisons using QUAST and BUSCO

To compare the assemblies in this study and the draft assembly, we used the genome comparison tool QUAST v5^25^ with the option – large and no reference assembly. QUAST was also run with the newly available chromosome level herring assembly as a reference. QUAST can also run a BUSCO analysis using the eukaryotic database. However, we chose to run a separate standalone BUSCO analysis using the Actinopterygii database^50^, to compare the completeness of the generated assemblies.

### Manual connexin analysis

A manual analysis of the connexin gene family^30^ was performed to assess the correctness and completeness of the assemblies. We collected all predicted connexin genes/mRNAs available in GenBank from the herring genome published by Martinez Barrio *et al*.^13^. This amounted to 49 connexin genes (before exclusion of near identical sequences). We also searched for additional (non-predicted) connexin genes in the published draft assembly using the NCBI Basic Local Alignment Search Tool (BLAST). Any hit was manually inspected, and two additional connexin sequences were found: one connexin gene predicted as *KAT6B-like* (a *gja8-like* sequence) and one previously non-predicted sequence (a *cx39*.*2*/*gjd2-like* sequence). After exclusion of five predicted sequences that showed >98.4% identity to other connexin sequences we had a set of 46 unique connexin sequences (Supplementary Table S2). We blasted the unique sequences against our unannotated assemblies and any unexpected hits were noted. Correspondingly, any unexpected hits in the published draft herring genome were noted.

### FRC

FRC^bam^ v1.3.0 and the paired-end and mate-pair data from the present study were used to evaluate the correctness of the assemblies^27^. The FRC^bam^ output consists of FRCs for 14 feature types. To rank the assemblies based on the different types of features, all 14 FRCs were plotted, and for each the best assembly was given 1 point, second best 2 points, and so on. If two assemblies had very similar curves, both assemblies received the same number of points. For example, A1 had the steepest curve and received 1 point, and both A2 and A3 had the second steepest curve so both received 2 points. Then, no assembly received 3 points, but the next assembly received 4 points. If the curve only had two points, the feature was excluded. The scores were summed and the assembly with the lowest score was ranked first.

Lastly, the assemblies were aligned against each other using D-Genies^37^ to determine whether any major structural variations existed.

## Supplementary information

Supplementary Tables and Figures

## Data Availability

The sequencing reads and assemblies are available in the European Nucleotide Archive repository, under the project accession http://www.ebi.ac.uk/ena/data/view/ERP107609.
