# Supplementary material for: Using long and linked reads to improve an Atlantic herring (Clupea harengus) genome assembly
Source: Sci Rep. 2019 Nov 27;9:17716. doi: 10.1038/s41598-019-54151-9 (PMC6881392; doi:10.1038/s41598-019-54151-9)
Supplement: Supplementary file 1 — Supplementary Tables and Figures [file 41598_2019_54151_MOESM1_ESM.pdf]

Supplementary Information

Title

Using long and linked reads to improve an Atlantic herring (*Clupea harengus*) genome assembly

Authors and institutional addresses

Sunnvør í Kongsstovu[1,2,4,*], Svein-Ole Mikalsen[2], Eydna í Homrum[3], Jan Arge Jacobsen[3], Paul Flicek[4], Hans Atli Dahl [1]

[1] Amplexa Genetics A/S, Hoyvíksvegur 51, FO-100 Tórshavn, Faroe Islands.

[2] University of the Faroe Islands, Dept. of Science and Technology, Vestara Bryggja 15, FO-100 Tórshavn, Faroe Islands.

[3] Faroe Marine Research Institute, Nóatún 1, FO-100 Tórshavn, Faroe Islands.

[4] European Molecular Biology Laboratory, European Bioinformatics Institute, Wellcome Genome Campus, Hinxton, Cambridge, CB10 1SD, UK.

***** Corresponding author email: skik@amplexa.com

Supplementary Table S1. The assembler, parameters and data used in the process of finding the optimal assembly (A1). The assembly that gave the best results is indicated with italics. PE indicates paired-end data and MP indicates mate-pair data.

**Assembler**	**Parameters**	**Data**
AllPaths-LG	Default + ploidy=2	160x PE and 37x MP
AllPaths-LG	Default + ploidy=2	120x PE[1] and 37x MP
AllPaths-LG	Default + haploidify=true + ploidy=2	90x PE[2] and 28x MP
AllPaths-LG	*Default + haploidify=true + ploidy=2*	*63x PE[2] and 37x MP*
AllPaths-LG	Default + ploidy=2	63x PE[2] and 37x MP
AllPaths-LG	Default + haploidify=true + ploidy=2	50x PE[3] and 37x MP
SGA	Default + k=41 + OL=75	160x PE and 37x MP
SGA	Default + k=31 + OL=65	160x PE and 37x MP
SGA	Default + k=31 + OL=75	160x PE and 37x MP
SGA	Default + k=31 + OL=85	160x PE and 37x MP
SGA	Default + k=51 + OL=65	160x PE and 37x MP
SGA	Default + k=51 + OL=75	160x PE and 37x MP
SGA	Default + k=51 + OL=85	160x PE and 37x MP
SGA	Default + k=61 + OL=75	160x PE and 37x MP
SGA	Default + k=61 + OL=85	160x PE and 37x MP
SGA	Default + k=71 + OL=30	160x PE and 37x MP
MaSuRCA	Default	160x PE, 37x MP and 2.4x MinION
Supernova	Default + bcfrac=0.5 + maxreads=300M + style=pseudohap	78.5x 10x Genomics
Supernova	Default + bcfrac=0.75 + maxreads=450M + style=pseudohap	78.5x 10x Genomics

[1] Only used the second run of the PE data.

[2] PE data selected on quality to only include 90x and 63x of the highest quality.

[3] PE data selected randomly.

Supplementary Table S2. Herring connexin genes. The predicted genes from the published herring genome assembly are indicated with a Genbank accession number, with the duplicate accession numbers in the comment section.

#	Abbreviated name	Accession number	Comments or Accession number of near duplicates (>98% id at nucleotide level)
1	gja1-cx43	XM_012829211	
2	gja1like	XM_012836783	
3	gja3like	XM_012842347	
4	gja3like	XM_012840585	
5	gja3like	XM_012834366	
6	gja3like	XM_012819598	
7	gja6like	XM_012822071	
8	gja5like	XM_012816449	
9	gja5like	XM_012840593	
10	gja8	XM_012840595	
11	NP-gja8	XM_012816450	Named as histone acetyltransferase KAT6B-like
12	gja9like	XM_012824682	
13	gja9like	XM_012816385	
14	gja10-cx62	XM_012821374	
15	gja10like	XM_012836705	
16	cx32.7like	XM_012829360	
17	cx32.2like	XM_012829221	
18	cx32.2like	XM_012829260	
19	cx32.2like	XM_012828709	
20	gjb1like	XM_012819602	
21	gjb2like	XM_012834339	
22	gjb2like	XM_012842299	
23	gjb2like	XM_012820173	
24	gjb2like	XM_012840586	
25	gjb3like	XM_012822385	100% identical to XM_012822374<br>100% identical to XM_012822365
26	gjb3like	XM_012818491	100% identical to XM_012818489
27	gjb4like	XM_012822073	
28	gjb4like	XM_012826764	
29	gjb4like	XM_012822396	
30	gjb4like	XM_012818492	99.9% identical to XM_012818490
31	gjb7-cx25	XM_012823856	
32	gjc1-cx45	XM_012816830	
33	gjc1like	XM_012817598	
34	gjc1like	XM_012821065	
35	gjc1like	XM_012836489	
36	gjc2-cx47	XM_012827872	
37	gjd2-cx36	XM_012823340	
38	gjd2	XM_012819299	
39	gjd2like	XM_012828866	
40	gjd2like	XM_012817227	

41	gjd2like	XM_012838313	
42	NP-cx39.2*		Identified by Blast using orthologs* from other teleosts
43	gjd3like	XM_012837668	98.4% identical toXM_012837669
44	gjd3like	XM_012837670	95.1% id to full length XM_012837668
45	gjd4-cx40.1	XM_012823059	
46	gje1like	XM_012822376	

* Sequences will be detailed elsewhere (Mikalsen SO, Tausen M, í Kongsstovu S, submitted).

Supplementary Figures

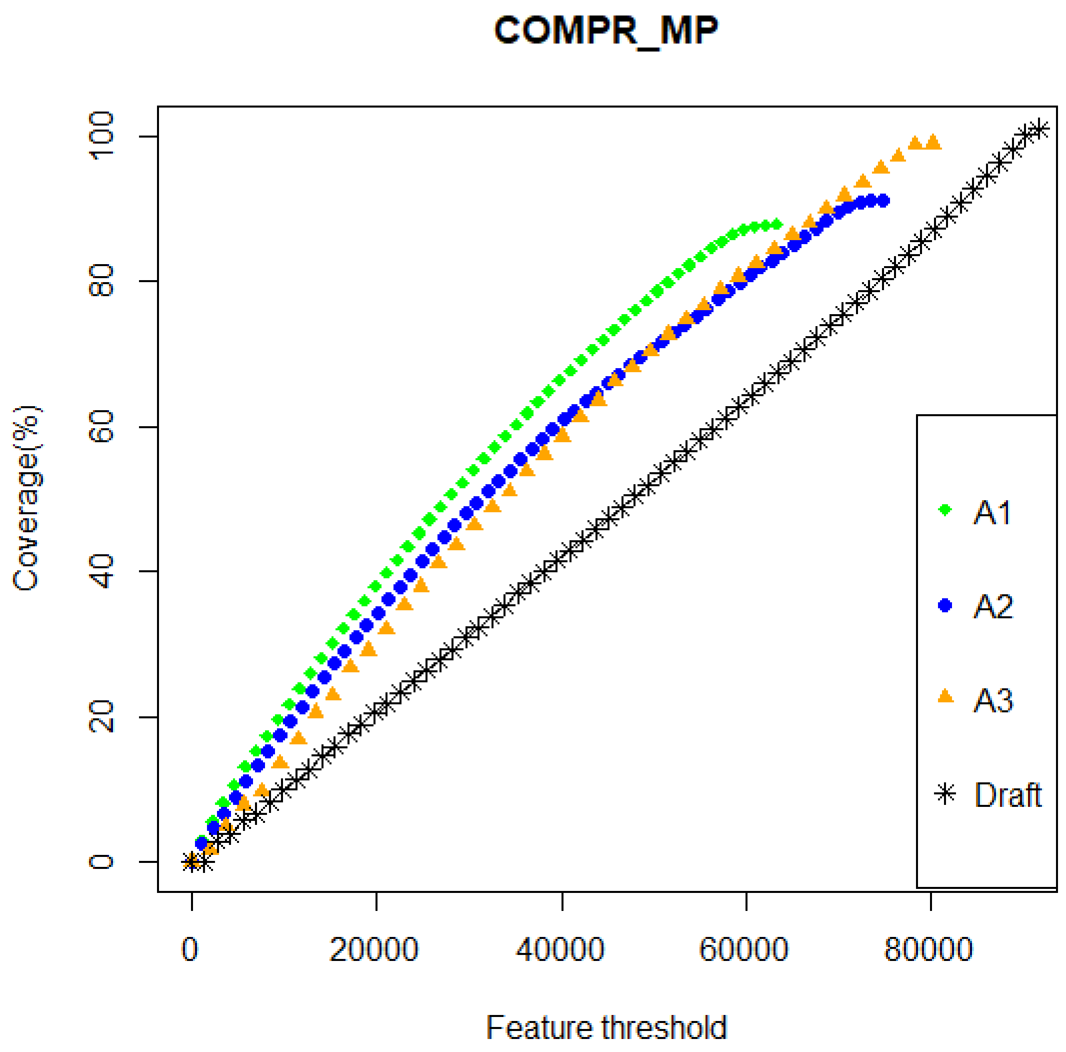

Supplementary Figure S1. Feature response curves (FRCs) for assemblies A1, A2, and A3 and the published draft herring assembly, showing COMPR_MP features. COMPR_MP features describe areas with low CE-statistics; that is, compressed sequences computed with mate-pair data[25]. The FRCs were generated using FRC[bam25] and plotted in R v3.4.3[50].

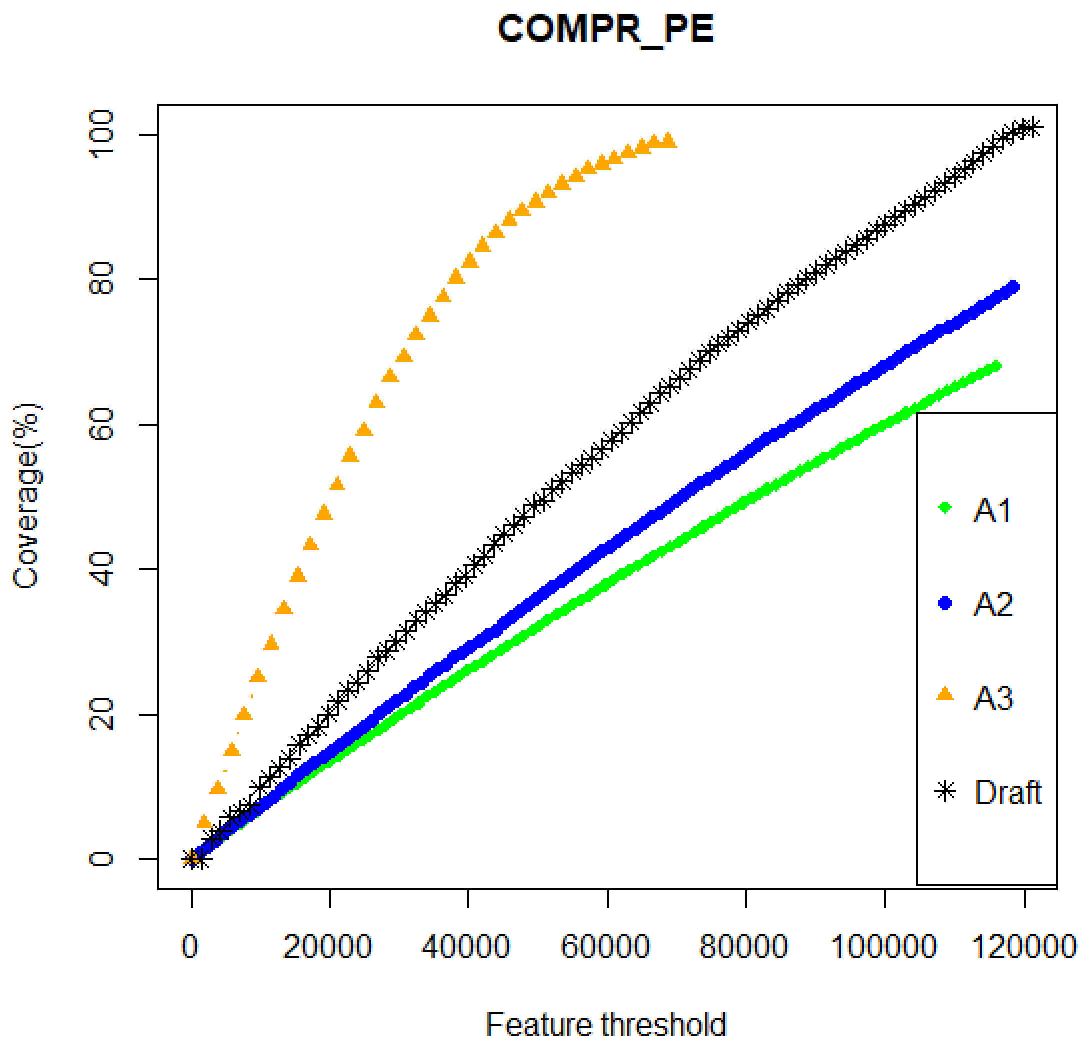

Supplementary Figure S2. Feature response curves (FRCs) for assemblies A1, A2, and A3 and the published draft herring assembly, showing COMPR_PE features. COMPR_PE features describe areas with low CE-statistics; that is, compressed sequences computed with paired-end data[25]. The FRCs were generated using FRC^{bam}[25] and plotted in R v3.4.3[50].

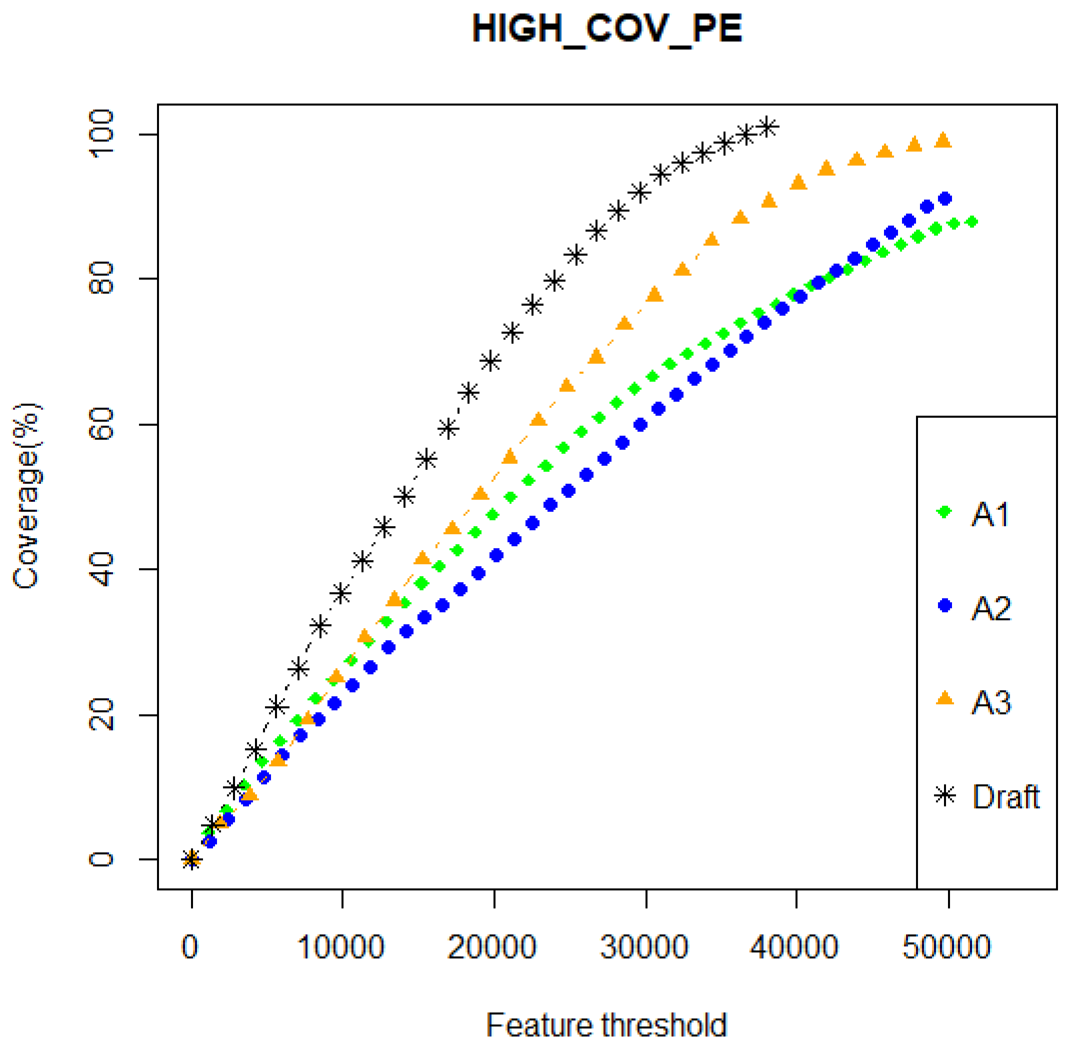

Supplementary Figure S3. Feature response curves (FRCs) for assemblies A1, A2, and A3 and the published draft herring assembly, showing HIGH_COV_PE features. HIGH_COV_PE features describe areas with high coverage, computed using all aligned reads[25]. The FRCs were generated using FRC[bam25] and plotted in R v3.4.3[50].

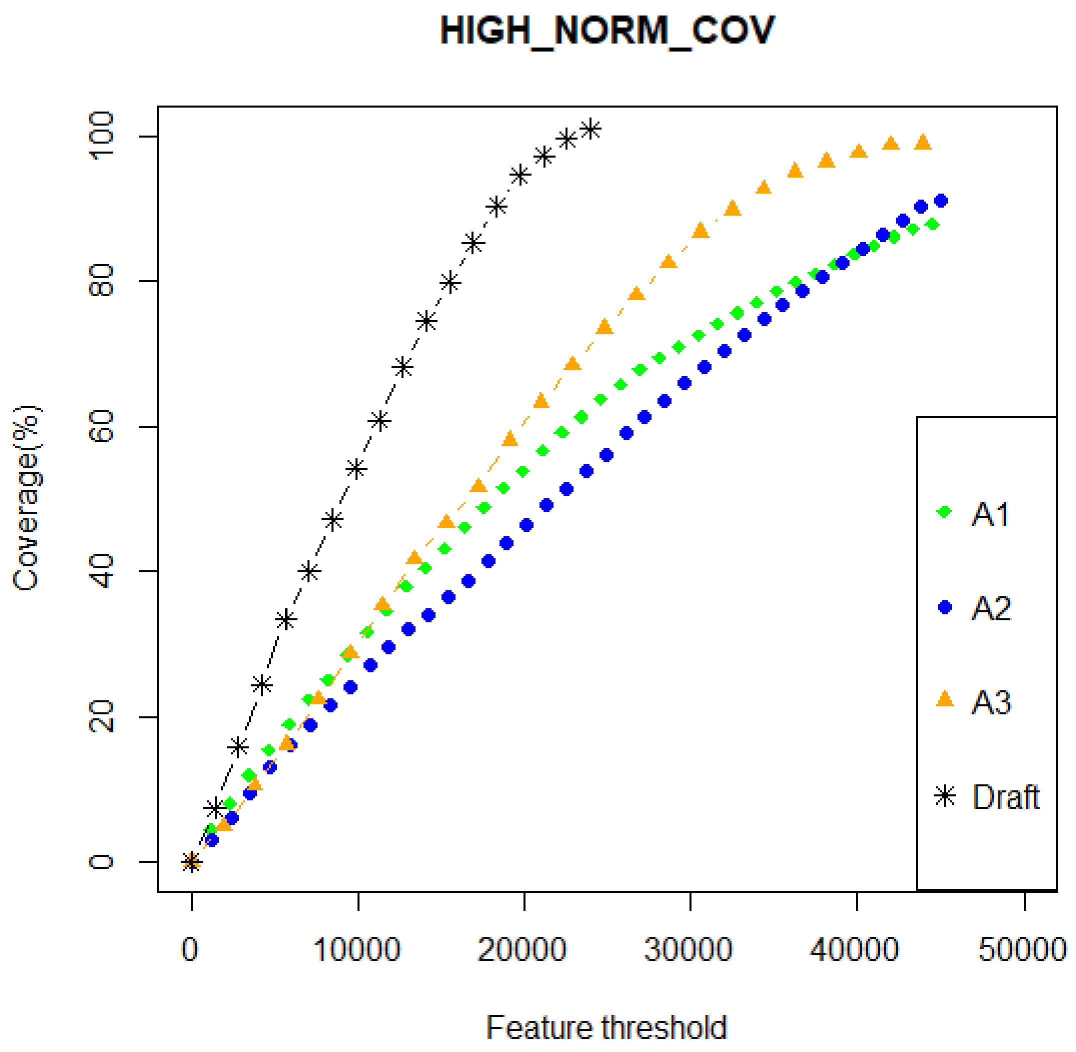

Supplementary Figure S4. Feature response curves (FRCs) for assemblies A1, A2, and A3 and the published draft herring assembly, showing HIGH_NORM_COV features. HIGH_NORM_COV features describe areas with high coverage, computed using only properly aligned read pairs[25]. The FRCs were generated using FRC[bam25] and plotted in R v3.4.3[50].

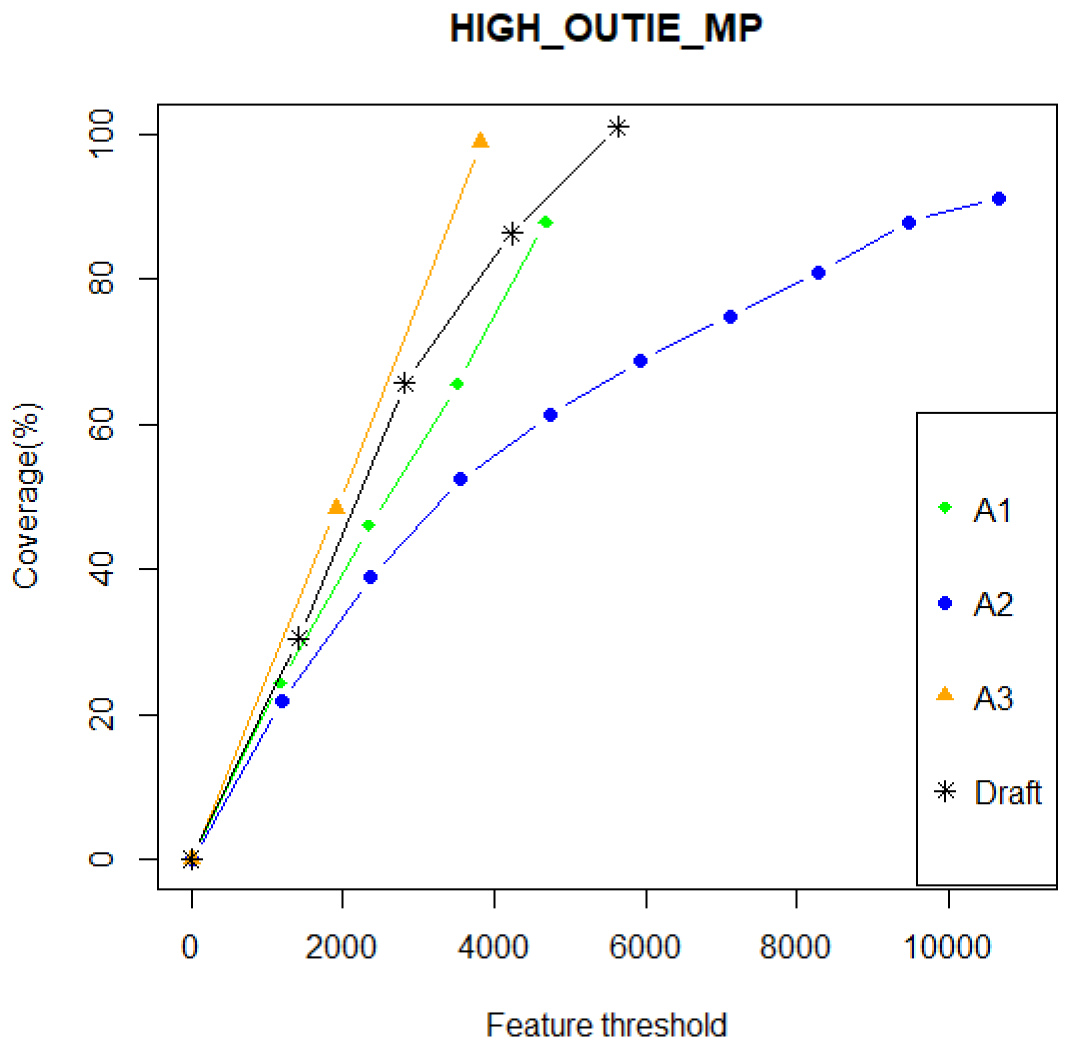

Supplementary Figure S5. Feature response curves (FRCs) for assemblies A1, A2, and A3 and the published draft herring assembly, showing HIGH_OUTIE_MP features. HIGH_OUTIE_MP features describe areas with a high number of mis-oriented or overly distant mate-pair reads[25]. The FRCs were generated using FRC[bam25] and plotted in R v3.4.3[50].

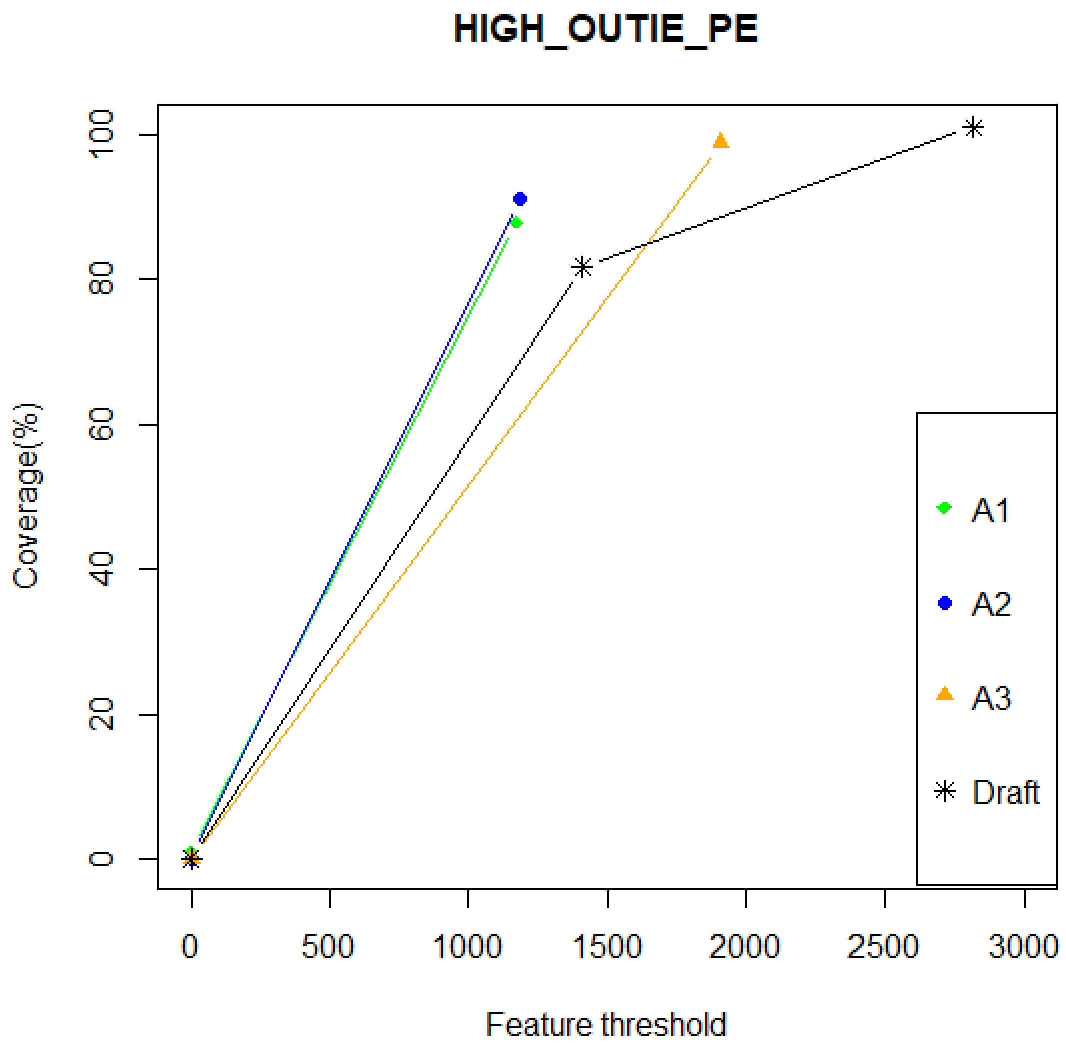

Supplementary Figure S6. Feature response curves (FRCs) for assemblies A1, A2, and A3 and the published draft herring assembly, showing HIGH_OUTIE_PE features. HIGH_OUTIE_PE features describe areas with a high number of mis-oriented or overly distant paired-end reads[25]. The FRCs were generated using FRC[bam25] and plotted in R v3.4.3[50].

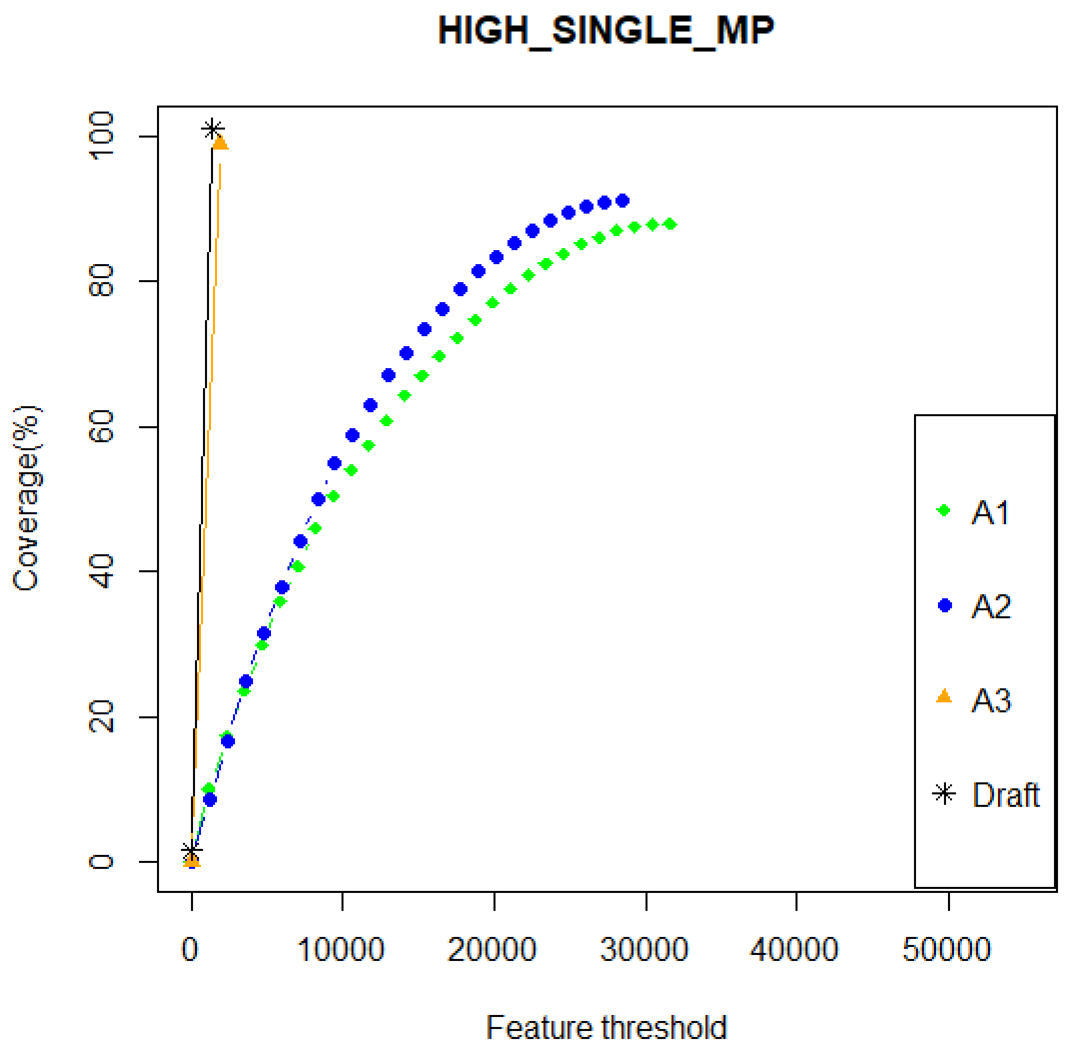

Supplementary Figure S7. Feature response curves (FRCs) for assemblies A1, A2, and A3 and the published draft herring assembly, showing HIGH_SINGLE_MP features. HIGH_SINGLE_MP features describe areas with a high number of mate-pair reads with unmapped pairs[25]. The FRCs were generated using FRC[bam25] and plotted in R v3.4.3[50].

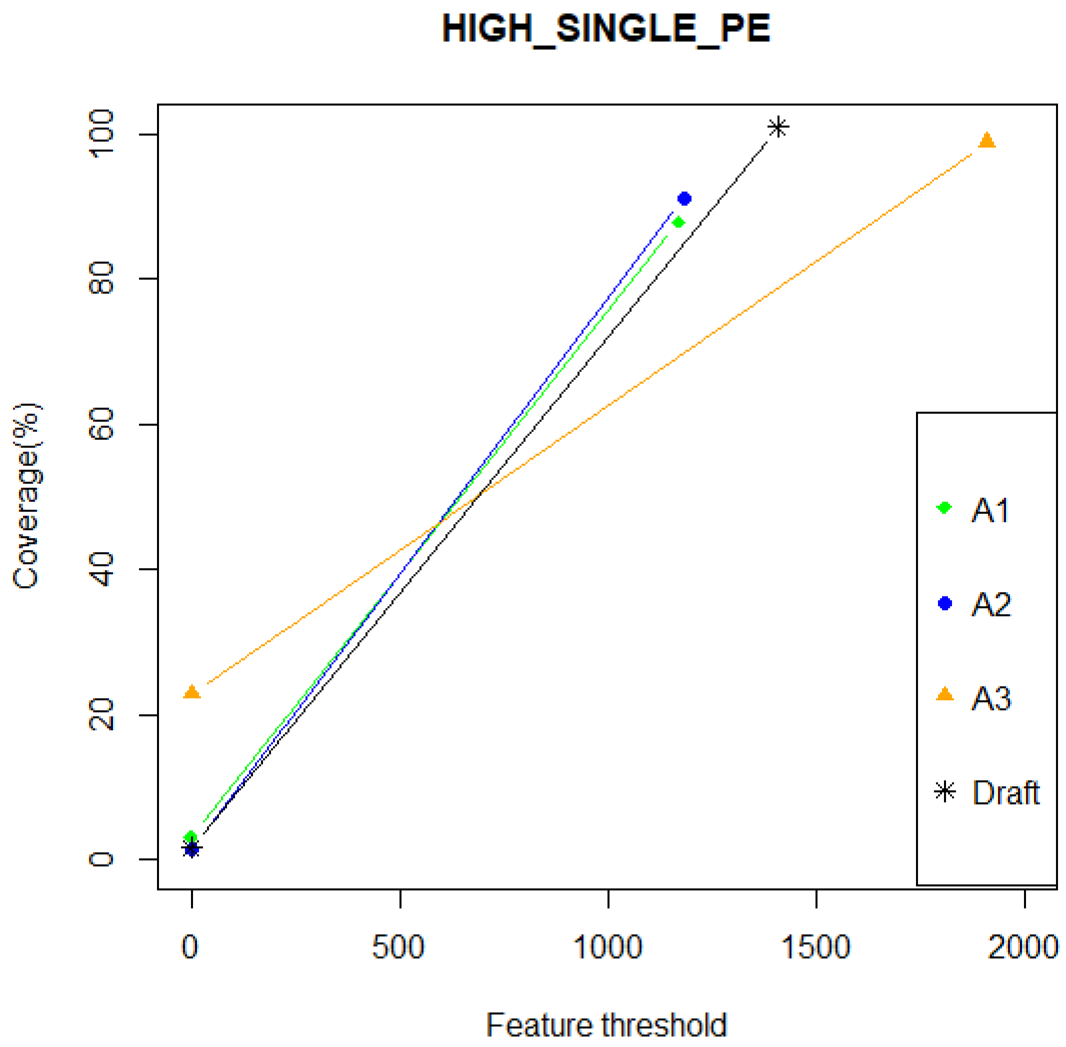

Supplementary Figure S8 Feature response curves (FRCs) for assemblies A1, A2, and A3 and the published draft herring assembly, showing HIGH_SINGLE_PE features. HIGH_SINGLE_PE features describe areas with a high number of paired-end reads with only one mapped read[25]. The FRCs were generated using FRC[bam25] and plotted in R v3.4.3[50].

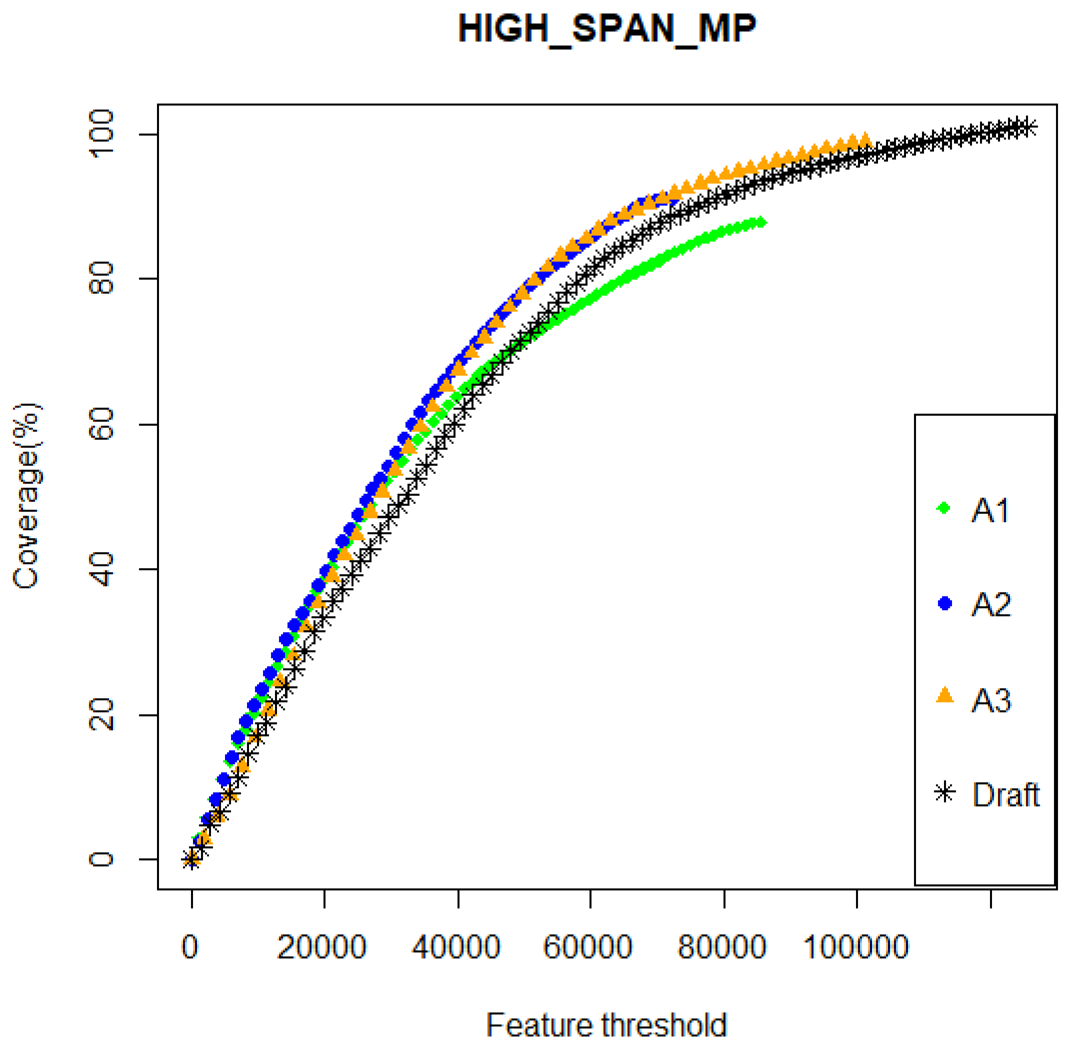

Supplementary Figure S9. Feature response curves (FRCs) for assemblies A1, A2, and A3 and the published draft herring assembly, showing HIGH_SPAN_MP features. HIGH_SPAN_MP features describe areas with a high number of mate-pairs mapping on different scaffolds[25]. The FRCs were generated using FRC^{bam}[25] and plotted in R v3.4.3[50].

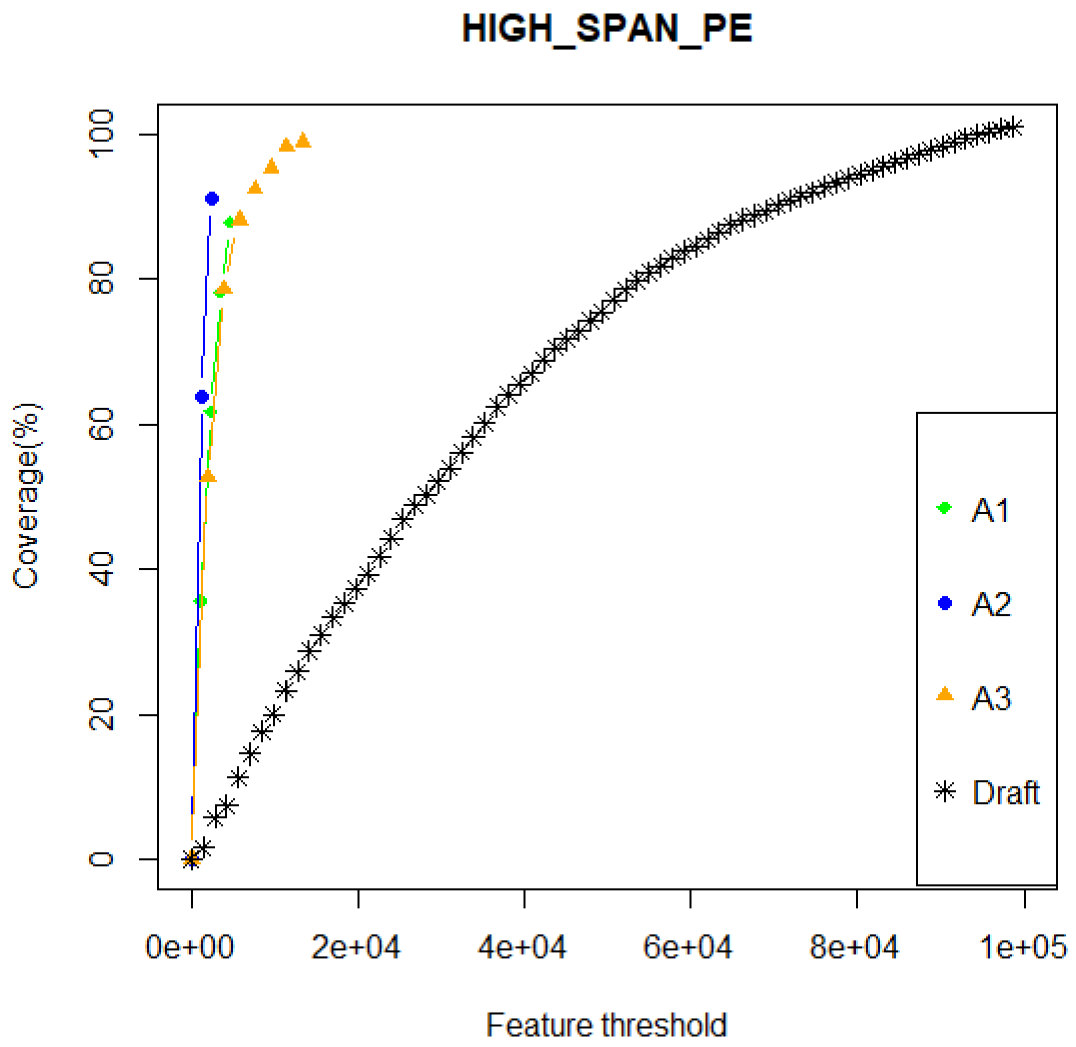

Supplementary Figure S10. Feature response curves (FRCs) for assemblies A1, A2, and A3 and the published draft herring assembly, showing HIGH_SPAN_PE features. HIGH_SPAN_PE features describe areas with a high number of paired-end reads mapping on different scaffolds[25]. The FRCs were generated using FRC^{bam}[25] and plotted in R v3.4.3[50].

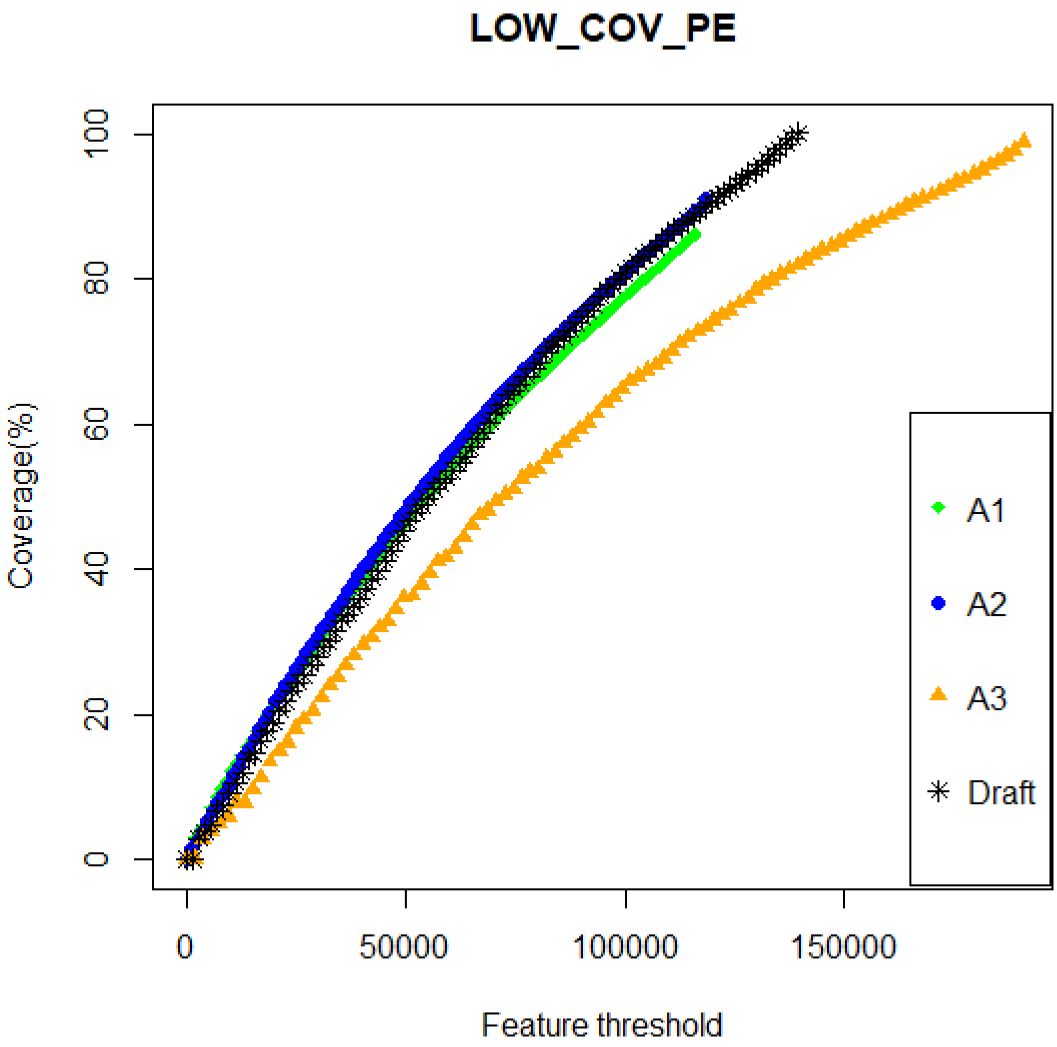

Supplementary Figure S11. Feature response curves (FRCs) for assemblies A1, A2, and A3 and the published draft herring assembly, showing LOW_COV_PE features. LOW_COV_PE features describe areas with low coverage, computed using all aligned reads[25]. The FRCs were generated using FRC[bam25] and plotted in R v3.4.3[50].

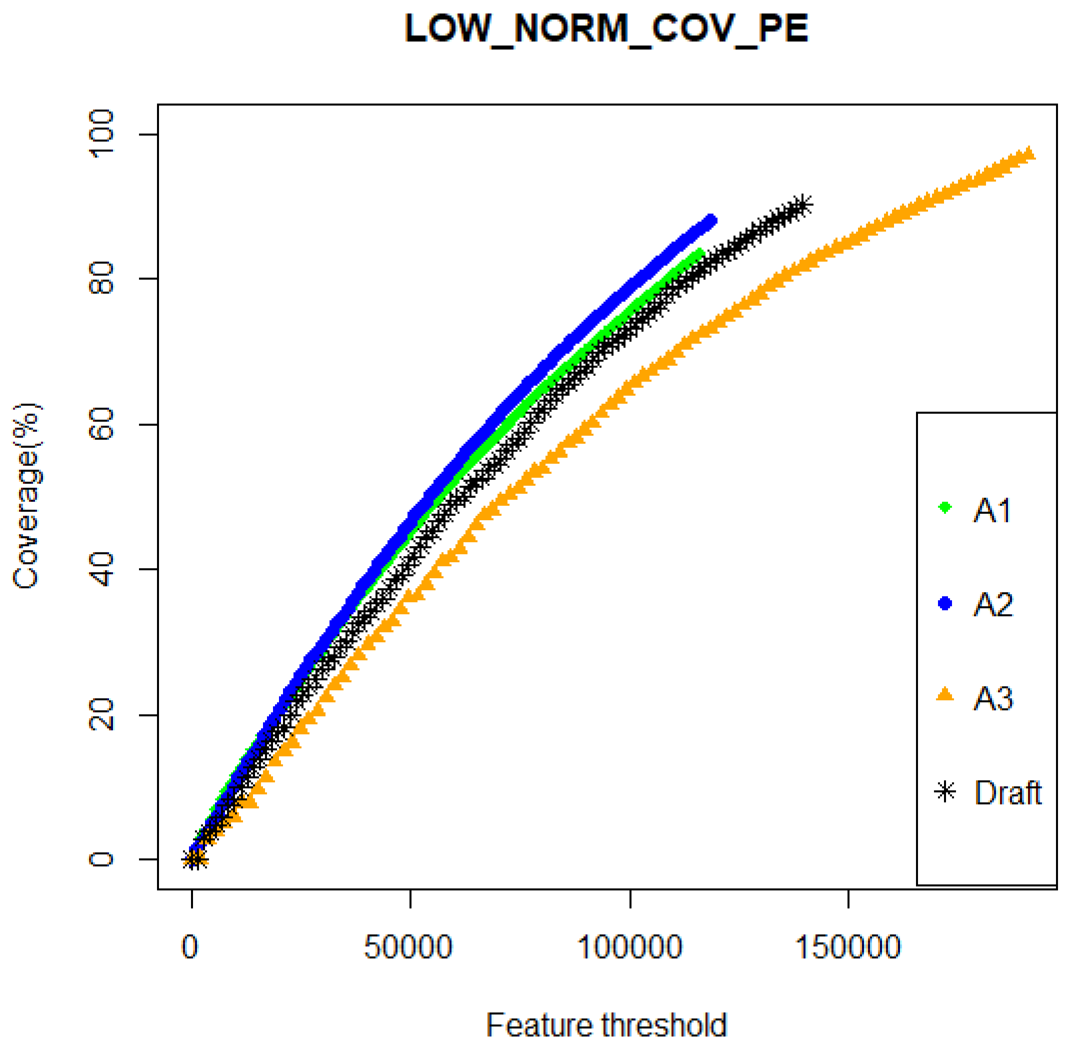

Supplementary Figure S12. Feature response curves (FRCs) for assemblies A1, A2, and A3 and the published draft herring assembly, showing LOW_NORM_COV_PE features. LOW_NORM_COV_PE features describe areas with low coverage, computed using only properly aligned pairs[25]. The FRCs were generated using FRC^{bam}[25] and plotted in R v3.4.3[50].

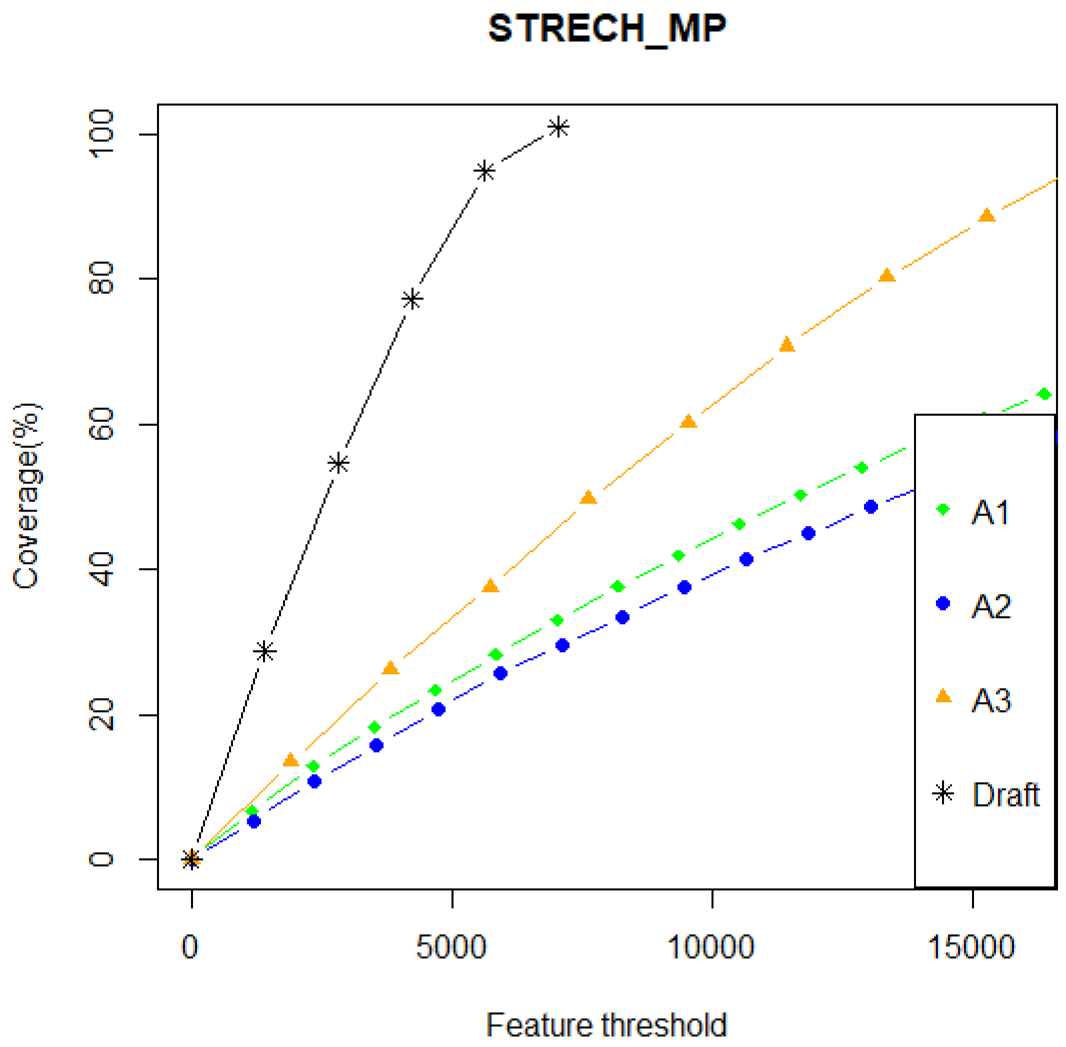

Supplementary Figure S13. Feature response curves (FRCs) for assemblies A1, A2, and A3 and the published draft herring assembly, showing STRECH_MP features. STRECH_MP features describe areas with high CE-statistics; that is, stretched sequences computed with mate-pair data[25]. The FRCs were generated using FRC^{bam}[25] and plotted in R v3.4.3[50].

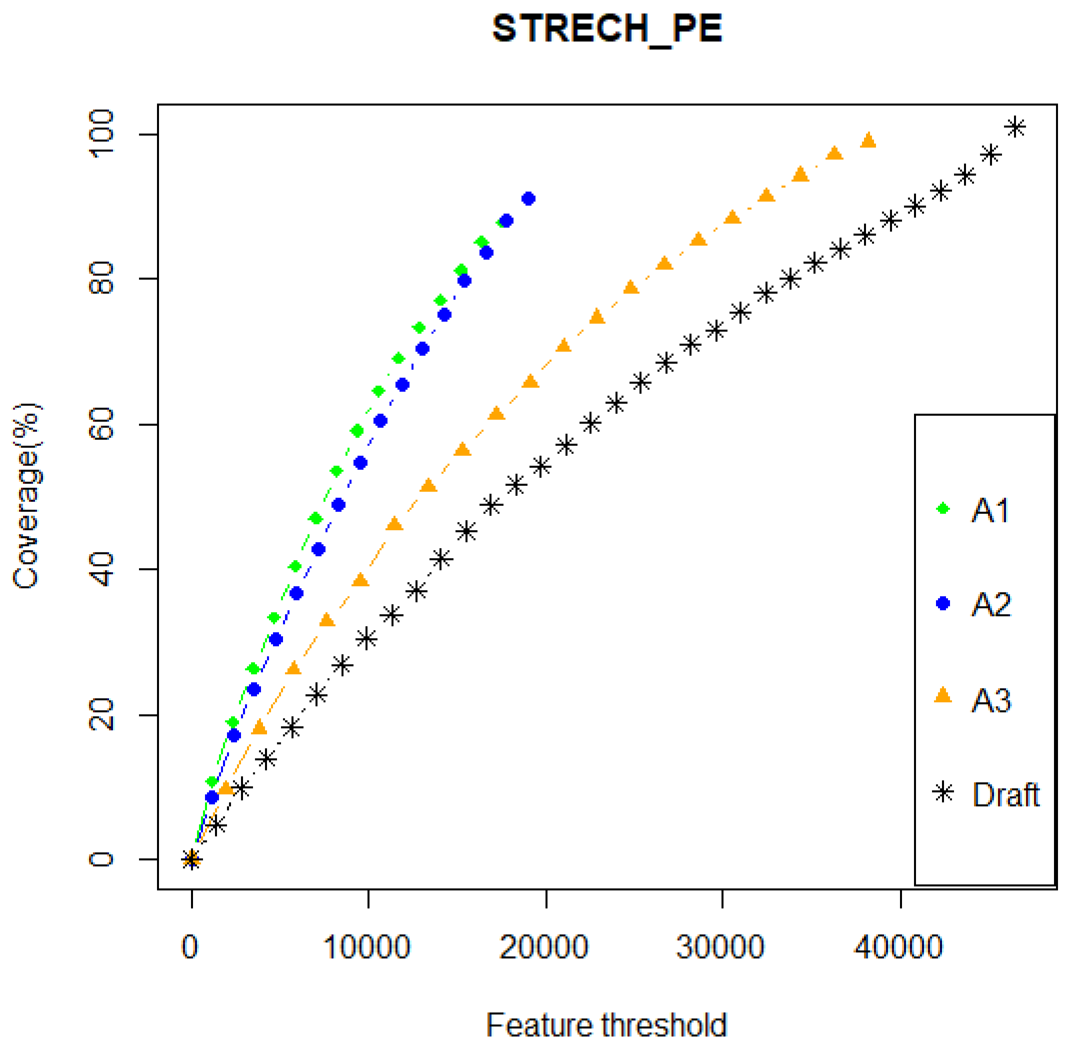

Supplementary Figure S14. Feature response curves (FRCs) for assemblies A1, A2, and A3 and the published draft herring assembly, showing STRECH_PE features. STRECH_PE features describe areas with high CE-statistics; that is, stretched sequences computed with paired-end data[25]. The FRCs were generated using FRC[bam25] and plotted in R v3.4.3[50].
